# How Surface Reconstruction Drives Heterogeneous Bulk Delithiation in Single‐Crystalline Ni‐Rich Layered Oxide Cathodes

**DOI:** 10.1002/smsc.70286

**Published:** 2026-05-12

**Authors:** Gaurav C. Pandey, Ashok S. Menon, Valeria Calani San Miguel, José J. Arroyo‐Gómez, Harry Gillions, Rebecca Sellers, Matthew J. W. Ogley, Eleni Fiamegkou, Satish Bolloju, Sahil Tippireddy, Mirian Garcia‐Fernandez, Steven Huband, Louis F. J. Piper

**Affiliations:** ^1^ Warwick Manufacturing Group (WMG) University of Warwick Coventry UK; ^2^ The Faraday Institution Quad One Harwell Science and Innovation Campus Didcot UK; ^3^ The Hartnoll Centre for Experimental Fuel Technologies University of Warwick Coventry UK; ^4^ Departamento de Almacenamiento de la Energía Subgerencia Operativa de Energía y Movilidad Instituto Nacional de Tecnología Industrial (INTI) Buenos Aires Argentina; ^5^ Consejo Nacional de Investigaciones Científicas y Técnicas (CONICET) Buenos Aires Argentina; ^6^ Diamond Light Source Ltd. Harwell Science and Innovation Campus Didcot UK; ^7^ Department of Physics University of Warwick Coventry UK

**Keywords:** Li‐ion battery cathodes, single‐crystalline Ni‐rich layered oxides, operando X‐ray diffraction, structural heterogeneity, surface reconstruction, intermittent current interruption

## Abstract

The electrochemical performance of single‐crystalline (SC) Ni‐rich layered oxide cathodes is fundamentally limited by bulk Li^+^ diffusion within micrometre‐sized particles. During high‐voltage cycling—necessary for high‐energy applications—intraparticle Li^+^ diffusion is further impeded by oxygen‐loss‐induced surface reconstruction from the layered phase to spinel/rock‐salt structures. Therefore, to fully understand how bulk Li^+^ transport kinetics influences electrochemical degradation, it is necessary to establish the correlation between surface reconstruction and bulk delithiation during the anisotropic structural evolution (i.e., expansion of the layers followed by their contraction) of the cathode particles during long‐term cycling. In this work, we accomplish this using multi‐rate operando X‐ray diffraction studies of SC Ni‐rich layered oxide cathodes aged under different voltage windows in single‐layer pouch full cells. We quantify how increased surface reconstruction leads to greater heterogeneity in bulk delithiation, thereby promoting phase separation and exacerbating electrochemical capacity fade. These results provide a direct mechanistic link between surface degradation and bulk delithiation in such cathodes and offer a framework for non‐destructively probing kinetics‐dependent degradation under practically relevant conditions to guide strategies for improved cycling stability.

## Graphical abstract

Through multi‐rate operando X‐ray diffraction and soft X‐ray absorption spectroscopy, we demonstrate how surface‐level changes in aged single‐crystalline Ni‐rich LiNi*x*Mn*y*Co_1−*x*−*y*_O_2_ particles can directly affect bulk Li‐ion diffusion. The effect of this phenomenon on the electrochemical performance is also quantified using single‐layer pouch cells under practically relevant operating conditions.

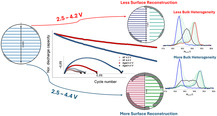

## 1 Introduction

Ni‐rich LiNi_*x*_Mn_*y*_Co_*z*_O_2_ (‘NMC’, where *x *+ *y *+ *z* = 1 and *x *≥ 0.8) compounds, are widely regarded as leading candidates for next‐generation lithium‐ion battery cathodes as they provide high energy density with reduced reliance on supply constrained cobalt [1, 2, 3]. These materials, with an *α*‐NaFeO_2_‐type structure and *R* $\bar{3}$ *m* space group symmetry, exhibit negative charge–transfer behaviour wherein delithiation is compensated by Ni 3*d*–O 2*p* rehybridisation, progressing from 3d^8^ to 3d^8^ L ^2^ (where L is a hole at O 2*p*) via ligand‐hole formation [4, 5, 6, 7]. At high states of delithiation, the ligand holes are unstable at the particle surface, and promote oxygen evolution, [8, 9, 10] which are compensated by transition‐metal (TM) migration and reduction [11, 12]. This drives a surface reconstruction from the layered structure to a denser cubic structure, forming the so‐called ‘spinel‐/rock‐salt‐like’ surface layer with a nominal composition of Li_*x*_ *TM*_1−*x*_O (where *TM* is Ni, Mn, and Co) [13, 14, 15, 16, 17, 18]. The degree of surface reconstruction depends strongly on the operational voltage window—charging above 4.3 V (vs. Li^+^/Li) increases oxygen‐vacancy formation and discharging below 3.0 V (vs. Li^+^/Li) promotes oxygen‐vacancy migration and densification of the surface layer [12, 19, 20, 21]. Furthermore, the released oxygen can also react with the electrolyte, generating gaseous by‐products (e.g., CO_2_ and CO) and solid decomposition products, which form the cathode–electrolyte interphase (CEI) on the surface [9, 22, 23, 24, 25]. Together, these surface phenomena impede Li^+^ transport across the electrode–electrolyte interphase and increase the overall cell impedance.

Although not directly linked to surface reconstruction, Ni‐rich layered oxide cathode particles undergo anisotropic expansion and contraction along the layer‐stacking direction during charge (delithiation)/discharge (lithiation) to/from voltages above 4.2 V (vs. Li^+^/Li). This is directly visible in the X‐ray diffraction (XRD) data in the evolution of the NMC 00*l* reflections during cycling, which can be tracked via the *c* lattice parameter of the NMC *R* $\bar{3}$ *m* unit cell. The repeated expansion–contraction over long‐term cycling can generate local stresses that progressively accumulate along grain boundaries and ultimately lead to particle fracture [26, 27, 28]. Polycrystalline cathodes, composed of secondary agglomerates of primary particles, are particularly susceptible to intergranular cracking, which exposes new particle surface area for O‐evolution‐induced surface reconstruction and parasitic cathode–electrolyte reactions, resulting in pronounced electrochemical capacity fade [26, 27, 28, 29]. Monolithic single‐crystalline (SC) particles (2–5 µm in size), which lack grain boundaries, are not generally vulnerable to mechanical degradation as they are more resilient to intergranular particle cracking [5, 17, 30, 31, 32, 33]. Nevertheless, they are still prone to changes at the particle surface that impede Li^+^ transport across the cathode–electrolyte interface, thereby increasing the cell impedance, and capacity fade [17]. The extent of surface transformation can vary among individual particles due to their anisotropic morphology and (crystallographic) facet‐dependent activity, [15] resulting in spatially inhomogeneous growth of surface layers. This leads to nonuniform (de)lithiation within individual particles as well as across the particle ensemble [34, 35], and is strongly influenced by the electrode design parameters such as the porosity, thickness, and cathode material volume fraction [36].

The reduced Li^+^ mobility induced by surface reconstruction can also influence the particle bulk, giving rise to the segregation of the overall particle into crystallographic domains with different Li^+^ concentration. These metastable phases predominantly form at high states of charge (SOC) and manifest in XRD data as the splitting of 00*l* reflections of cathode (NMC811) phase towards lower scattering angles [30, 37, 38]. This is further exacerbated by the intrinsically longer diffusion pathways in micron‐sized SC particles. This bulk segregation of the cathode bulk into spatially inhomogeneous domains with different Li^+^ concentrations, and by extension, electrochemical activity, is commonly referred to as structural fatigue. Over the course of cycling, the effects of these surface and bulk changes compound, resulting in a highly fatigued bulk structure, and lead to notable kinetically‐dependent capacity fade (i.e., influenced by the cycling rate) [34, 39, 40, 41, 42]. This cycling‐rate‐dependent capacity fade is a major bottleneck for the further development of SC Ni‐rich layered oxides for industrial applications. Surface engineering techniques like atomic layer deposition have been demonstrated to be effective in mitigating deleterious changes at the particle surface, thereby improving overall Li^+^ mobility and electrochemical performance [43]. Taken together, the electrochemical performance of SC Ni‐rich layered oxide cathodes is governed by the interplay between particle surface transformations and their impact on the bulk structure. This is, in turn, strongly dependent on operating conditions, most notably the cycling rate and voltage window. Consequently, parameterisation of rate‐dependent degradation and evaluation of mitigation strategies require diagnostic methodologies capable of directly probing the correlation between these factors in a statistically relevant manner at the electrode/cell level under representative operating conditions. This is nontrivial, as correlations between surface reconstruction, electrochemical performance, and bulk structural heterogeneity are typically established ex situ using techniques such as soft X‐ray absorption spectroscopy, X‐ray photoelectron spectroscopy, or transmission electron microscopy, which often cannot capture the real‐time evolution of the intermediate metastable cathode states.

Although the chemical nature of the surface reconstruction layer, its Li‐ion transport properties and contribution to capacity fade, and the existence of bulk structural fatigue upon electrochemical ageing have already been demonstrated for SC Ni‐rich layered oxide cathodes, a direct quantitative investigation of the correlation between these phenomena is lacking. In other words, to what extent does surface reconstruction quantitatively modulate bulk delithiation pathways during cycling, and how does it correlate with long‐term degradation? In this work, we demonstrate and explain how this correlation evolves as a function of operational voltage windows and cycling rate in SC Ni‐rich layered oxide cathodes, which can be investigated in operando using multi‐rate operando XRD of single‐layer pouch full cells. Different degrees of cathode surface reconstruction were induced by cycling the cells to upper cut‐off voltages below and above the oxygen evolution threshold of the cathode, 4.2 and 4.4 V (vs. graphite), respectively. Surface reconstruction is exacerbated through a combination of a lower cut‐off voltage of 2.5 V and an elevated cycling temperature of 40°C. The ageing protocols were engineered such that, after 100 C/3 cycles, both cells exhibited comparable specific capacities. This enabled a direct investigation of the extent to which kinetics‐dependent capacity fade is governed by bulk and surface cathode modifications induced by cycling across the 2.5–4.4 V voltage window. Soft X‐ray absorption spectroscopy and XRD measurements confirm that structural changes at this stage of ageing are predominantly confined to the particle surfaces (<10 nm). The resulting impact of surface reconstruction on bulk structural transformations is then directly tracked and quantified using multi‐rate operando XRD. This approach enables a direct assessment of how surface reconstruction governs bulk (de)lithiation behaviour and, by extension, the electrochemical performance, thereby providing mechanistic insights that are essential for translating fundamental understanding into designing and evaluating practical strategies to mitigate degradation.

## 2 Results and Discussion

### 2.1 Effect of Upper Cut‐Off Voltage on Electrochemical Performance

**FIGURE 1:**
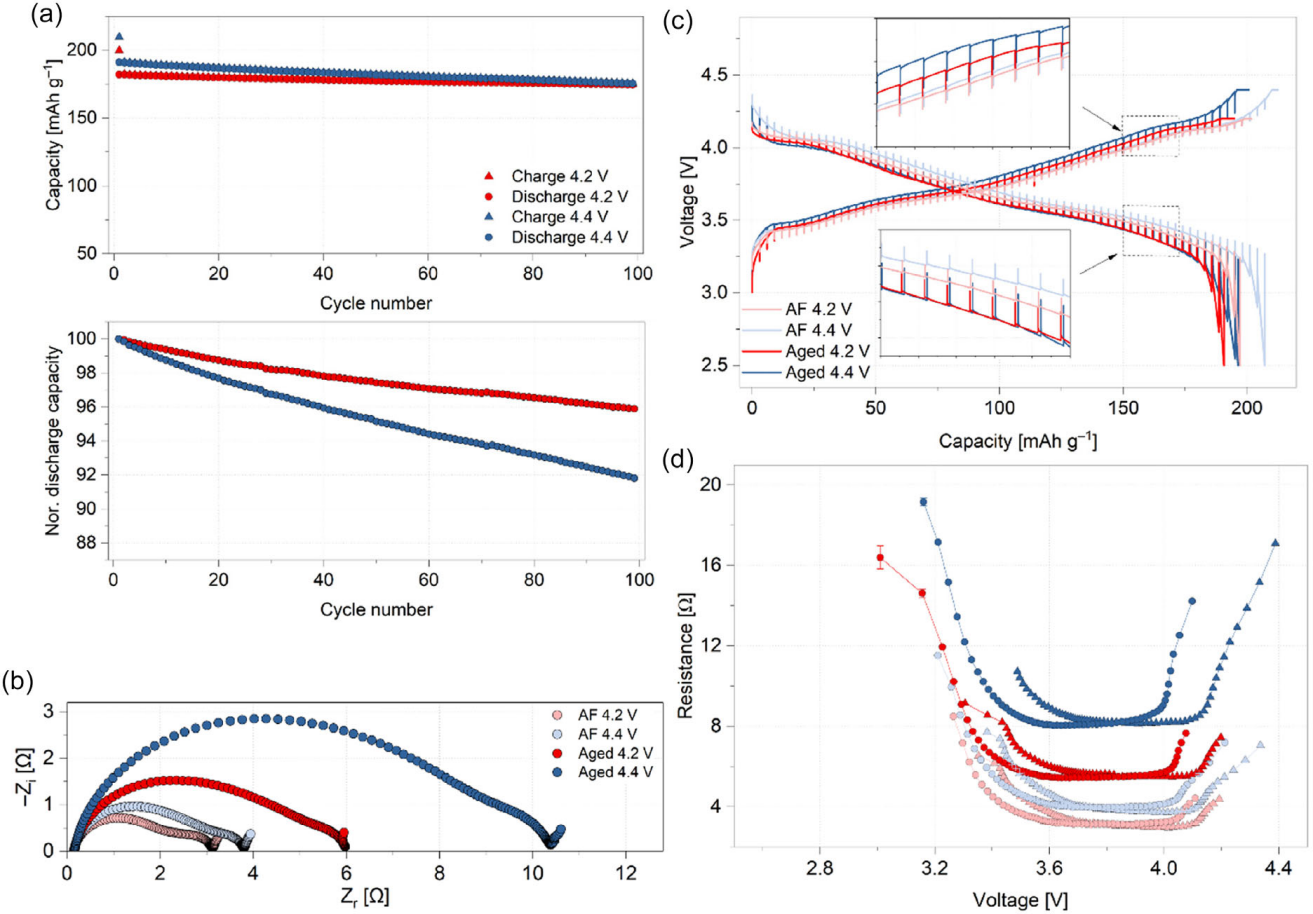
Electrochemical cycling data of the single‐layer pouch cells within the 2.5–4.2 V (red) and 2.5–4.4 V (blue) voltage windows. (a) Absolute and normalised (nor.) specific capacities as a function of cycle numbers (C/3 rate). (b) Nyquist plots of the electrochemical impedance spectroscopy data from the cells, measured at 3.8 V (AF–after formation cycles, aged–after 100 C/3 cycles). (c) Voltage versus capacity profiles of diagnostic C/10 cycles with intermittent current interruption, with an enlarged view of the voltage spikes in insets. (d) The corresponding internal resistance profiles determined from the analysis of the intermittent current interruption data. The legend in (a) also applies to (d).

The phase purity and SC morphology of the Li_1.01_Ni_0.81_Mn_0.06_Co_0.11_O_2_ (henceforth referred to as NMC811 for convenience) cathode particles, identical to those used in our previous work, were verified by powder XRD data and scanning electron microscopy (SEM), respectively. All electrochemical cycling and testing were done in single‐layer pouch cells (∼70 mAh, ∼2.1 mAh cm^−2^) against a graphite negative electrode. As mentioned, different degrees of surface reconstruction on the NMC811 cathode particles were induced by electrochemically ageing the cells to different upper cutoff voltages (4.2 and 4.4 V) for 100 cycles at C/3 rate. Hereafter, they are referred to as the 4.2 and 4.4 V cells. Before electrochemical ageing, both cells underwent two formation cycles at a rate of C/20 within their respective voltage windows (Supporting Information, Table S1 and Figure S1). During the first formation cycle, the 4.2 and 4.4 V cells exhibited relatively low coulombic efficiencies, 85.6% and 93.7%, respectively. This is primarily due to loss of Li^+^ inventory associated with the formation of the solid–electrolyte interphase (SEI) layer on the graphite anode [44]. The higher efficiency of the 4.4 V cell indicates the larger extent of Li^+^ (de‐)intercalation. In the second formation cycle, both cells achieved coulombic efficiencies close to 99%, suggesting that the electrode–electrolyte interfaces are largely stabilised after the initial cycle [45]. After the formation cycles, a C/10 diagnostic cycle with intermittent current interruption (ICI) protocol was performed on each cell to track the evolution of internal resistance as a function of voltage [46]. Following this, the cells were aged at a C/3 rate using constant‐current–constant‐voltage (CCCV) charge and CC discharge. In the first C/3 cycle, both cells exhibited a low coulombic efficiency of ∼91%, which recovered to >99% by the second C/3 cycle and thereafter remained stable until the last C/3 cycle (Figure S1b). Although the 4.4 V cell delivered a higher capacity than the 4.2 V cell initially, this advantage gradually diminished with continued cycling. By the 100^th^ cycle, both cells converged to comparable charge and discharge capacities of ∼175 mAh g^−1^. Relative to the second C/3 cycle, the 4.2 V cell retained 95.8% of its discharge capacity, a 4.2% loss, while the 4.4 V cell retained only 91.9%, an 8.15% loss (Figure 1a) [30]. Following the ageing cycles, a C/10 diagnostic cycle yielded unusually high coulombic efficiencies (∼110%) for both cells, contrary to the low efficiencies (∼91%) seen in the first C/3 cycle (Figure S1b). This anomaly arises from incomplete delithiation of the graphite anode during the faster C/3‐rate discharges, which is recovered during the slower C/10 cycle [47]. Similar post‐cycling capacity recovery behaviour under slow rate conditions was also observed in our earlier works [30, 43]. Another diagnostic cycle with the ICI protocol was also performed to quantify the voltage‐dependent resistance trends after ageing.

Figure 1b shows the electrochemical impedance spectroscopy (EIS) data of the 4.2 and 4.4 V cells as Nyquist plots, recorded at 3.8 V after the formation cycles and following the ageing. After formation, the overall impedance of the 4.4 V cell was slightly higher than that of the 4.2 V cell. After 100 cycles, the gap between the two cells became more pronounced, with the 4.4 V cell exhibiting substantially higher impedance. This difference is attributed to the detrimental changes at the cathode particle surface due to charging to higher voltage, consistent with the oxygen‐loss driven surface reconstruction and associated parasitic side reactions. The EIS data were fitted using the equivalent circuit model (ECM) shown in Figure S2, and the refined fitting parameters are summarised in Table S2, which shows that surface/SEI‐ and charge–transfer related resistances are the dominant contributor to impedance rise, particularly in the 4.4 V cell.

To understand the link between the operational voltage window, surface reconstruction and bulk (de)lithiation, it is necessary to understand how the internal resistance of the cells (i.e., sum of resistive contributions that follow Ohm's law) evolves as a function of the voltage (and state of charge). This can be obtained from the diagnostic ICI cycles (Figure 1c). During ICI cycling, the applied current is periodically interrupted, which produces an instantaneous voltage spike/drop as the cell relaxes to its equilibrium voltage, as illustrated in the enlarged insets of Figure 1c. The trends observed for the two cells are analogous to those observed for the EIS data. The 4.4 V cell shows higher voltage spikes than the 4.2 V cell during both the after‐formation and aged states, with the difference between the cells becoming more pronounced after ageing. The resistance evolution at the after‐formation and aged states are shown in Figure 1d. The 4.4 V cell displayed slightly higher overall resistance than the 4.2 V cell throughout the cycle after formation, but this difference increases substantially after ageing. As shown in Figure 1d, the internal resistance is highest near the fully delithiated and fully lithiated states, i.e., at high and low SOC. In the delithiated regime, the sharp increase in resistance is most likely associated with the previously discussed structural contraction of the inter‐layer distances, which shrinks the effective Li^+^ diffusion pathways. At high degrees of lithiation, further Li^+^ insertion becomes kinetically constrained due to the low availability of lithium vacancies. In contrast, the voltage window between 3.7 and 4.1 V represents the most kinetically favourable regime for Li^+^ (de‐)intercalation [48]. This is corroborated by the Li^+^ diffusivity trends reported elsewhere, which show a similar drop in values towards the beginning and end of charge and discharge [36]. The nature of the resistance increase in the 4.4 V cell markedly differs between this kinetically favourable 3.7–4.1 V region and the voltage extremes. Within the 3.7–4.1 V window, an almost rigid shift in resistance is observed between the after‐formation and aged states. However, toward the end of delithiation, the internal resistance in the aged cell increases much more steeply than in the after‐formation state. This behaviour cannot be attributed solely to oxygen‐loss‐driven surface reconstruction and/or parasitic side reactions associated with the higher upper cut‐off voltage, as such processes would be expected to affect the resistance across the entire voltage range rather than predominantly at high potentials. Instead, this suggests a coupled effect of surface transformations and structural transformations that together give rise to the pronounced resistance increase at high states of delithiation. A comparable behaviour is not observed in the 4.2 V cell.

The effect of the different cycling windows on the particle microstructure and cracking was investigated through cross‐sectional SEM. As seen in Figure S3, both cathodes show no evidence for cracking in the aged cathodes, indicating that particle cracking does not influence their electrochemical performance. This suggests that the observed electrochemical trends arise predominantly due to changes in the cathode material properties at the surface and bulk. The correlations between these changes will be explored in the next sections.

### 2.2 Surface Versus Sub‐Surface/Bulk Changes in Aged NMC811 Cathodes

**FIGURE 2:**
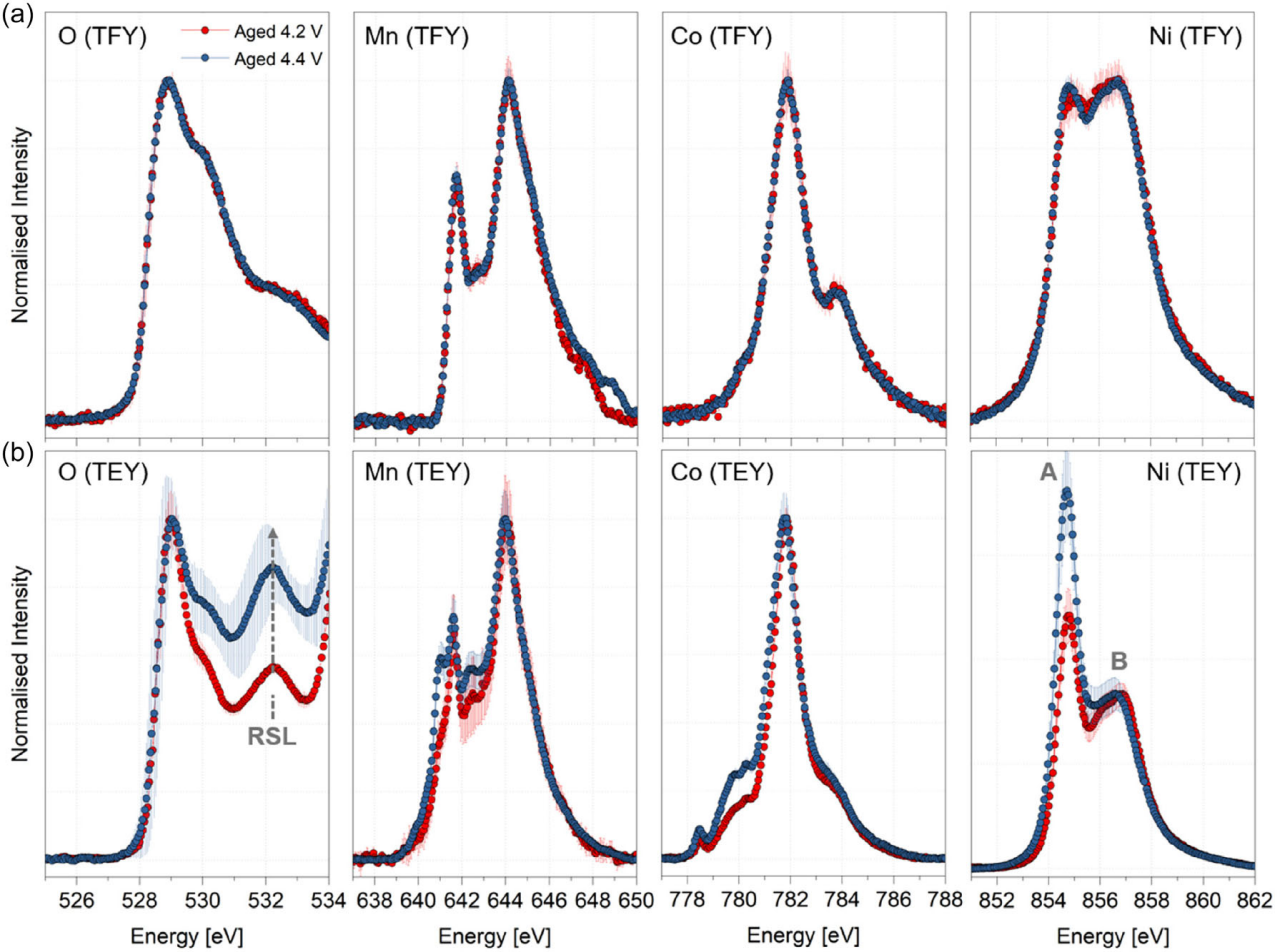
Background‐subtracted and normalised soft XAS spectra for aged 4.2 and 4.4 V cathodes in the discharged state. (a) O K‐edge pre‐peak and Mn, Co, and Ni, L_3_‐edge spectra collected in sub‐surface sensitive TFY mode. (b) Corresponding spectra collected in surface‐sensitive TEY mode.

Figure 2 shows the soft X‐ray absorption spectra collected at the O K‐edge and the Mn, Co, and Ni L_3_‐edges for the aged 4.2 and 4.4 V cathodes in their discharged state, measured in both sub‐surface sensitive total fluorescence yield (TFY, probe depth 100–200 nm) and surface sensitive total electron yield (TEY, probe depth 1–10 nm) modes. In all cases, the data shown is the average of spectra collected from three distinct locations on the cathode. At the O K‐edge, the pre‐edge feature around ∼528 eV arises due to the electronic transitions from the O 1*s* to the TM 3*d*–O 2*p* hybridised states, whereas the Mn, Co, and Ni L_3_‐edges originate from dipole‐allowed 2*p* to 3*d* electronic transitions [4, 49]. As illustrated in Figure 2a, the TFY spectra for all elemental edges overlap for both aged cathodes, indicating that the extent of sub‐surface cathode oxidation/reduction is very similar. Relative to the pristine data (Figure S4), the aged cathodes exhibit a higher degree of oxidation in the sub‐surface, as expected from irreversible bulk Li^+^ loss over the course of cycling. Further, the O K‐edge resonant inelastic X‐ray scattering (RIXS) data of the aged cathodes, collected at two incident energies of 531.0 and 531.2 eV, are shown in Figure S5. The data are highly comparable, signifying that the underlying oxygen‐related electronic excitations remain essentially unchanged in the sub‐surface region. This is consistent with the aforementioned TFY data. Very weak vibronic features are observed close to the elastic line, but the data lack sufficient statistical confidence for any definitive interpretation.

In contrast, the surface‐sensitive TEY spectra reveal clear differences between the two aged cathodes, as shown in Figure 2b. In the O K‐edge data, in addition to pre‐edge peak at ∼528 eV, an additional feature at ∼532 eV is observed, which is absent in TFY mode and widely associated with the formation of ‘NiO‐like (rock salt)’ surface layer in Ni‐rich layered oxides [4, 50]. This feature is more pronounced in the aged 4.4 V cathode, indicating a greater extent of surface reduction. However, the presence of both the pre‐edge and the 532 eV features in the spectra implies that the extent of surface reconstruction is thinner than the TEY probing depth (1–10 nm), consistent with previous reports [13, 51, 52]. No evidence of Li_2_CO_3_ formation is observed, as the characteristic ∼534 eV peak is absent. At the Mn and Co L_3_‐edges in TEY mode, the aged 4.4 V cathode shows more reduction, evidenced by the low‐energy features at 641–642.5 eV for Mn and ∼780 eV for Co [53, 54]. Further evidence for reduction is also observed in the Ni L_3_‐edge data, as evidenced by the relative integral ratio of the two peaks denoted by *A* and *B*. Following previous studies, the comparatively lower *B*/*A* integral ratio of the 4.4 V cathode can be used as an indicator for a more reduced state at the surface [4]. This is consistent with the stronger ‘rock salt’ signature observed at the O K‐edge [4]. Overall, the TEY data unequivocally indicate that the surface of the aged 4.4 V cathode is more reduced than that of the aged 4.2 V cathode. For reference, the corresponding spectra of the pristine cathode are provided in Figure S4. The full‐range soft XAS data are shown in Figures S6 and S7.

Further confirmation that the cathode bulk remains largely unchanged is obtained from XRD measurements of the two aged cells in the discharged state (Figure S8). Consistent with the subsurface‐sensitive soft XAS results, the XRD patterns show no significant differences between the cells apart from minor peak shifts; the cathode and anode peak profiles are otherwise highly comparable. To determine how the aforementioned surface changes influence delithiation in the two cathodes, operando XRD measurements were performed on the same cells.

### 2.3 Surface–bulk Correlation via Multi‐Rate Operando XRD

**FIGURE 3:**
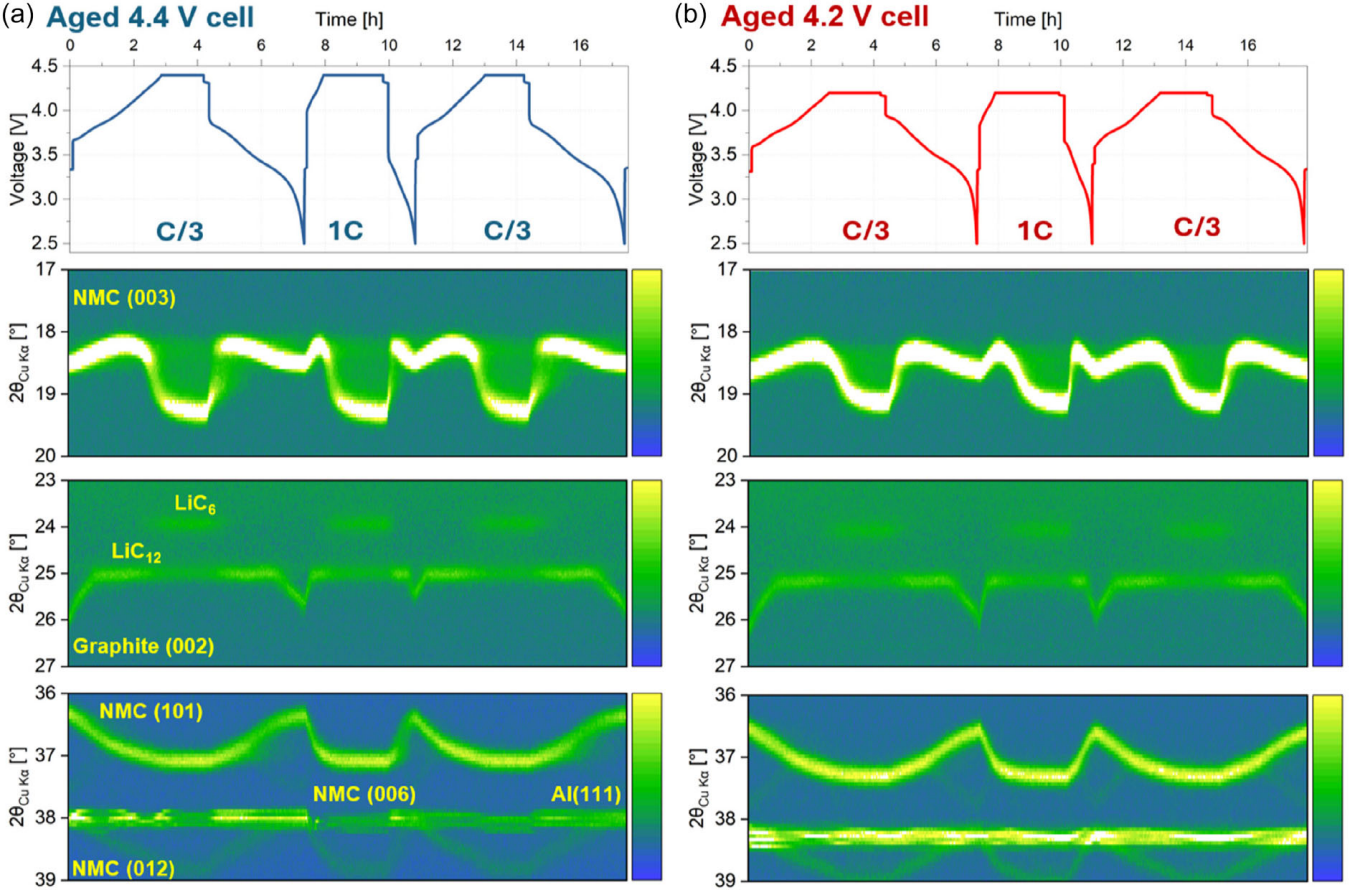
Operando XRD heatmaps for aged NMC811–graphite pouch cells cycled within (a) 2.5–4.4 V and (b) 2.5–4.2 V voltage windows. Each diffraction pattern was collected for a duration of 120 s. The corresponding voltage–time profiles are shown above each heatmap.

The dependence of bulk (de)lithiation on the degree of surface reconstruction in the aged NMC811 cathodes was investigated through multi‐rate operando XRD measurements. Figure 3 presents heatmaps of the operando XRD data collected during three consecutive cycles: C/3, 1C, and C/3, with the corresponding voltage–time profiles shown on top. Each cycle had a constant‐voltage (CV) hold (cutoff at one‐tenth of the current value) and 10 min. rest (*I* = 0 mA), in that order, between the constant‐current (CC) charge and discharge steps. The diffraction data include contributions from the (003), (101), and (012) reflections of the NMC811 phase (*R* $\bar{3}$ *m* space group symmetry). The graphite (002) reflection with *P*6_3_/*mmc* space group symmetry is also observed, as well as those of the lithiated phases, LiC_12_ (*P*6_3_/*mmc*) and LiC_6_ (*P*6/*mmm*). The (111) reflection from the aluminium current collector (*Fm* $\bar{3}$ *m*) is also seen. The position of this reflection is invariant during the cycles, confirming that the observed shifts in NMC811 and graphite reflections arise from structural changes rather than extraneous sample/cell displacement or instrumental effects.

**FIGURE 4:**
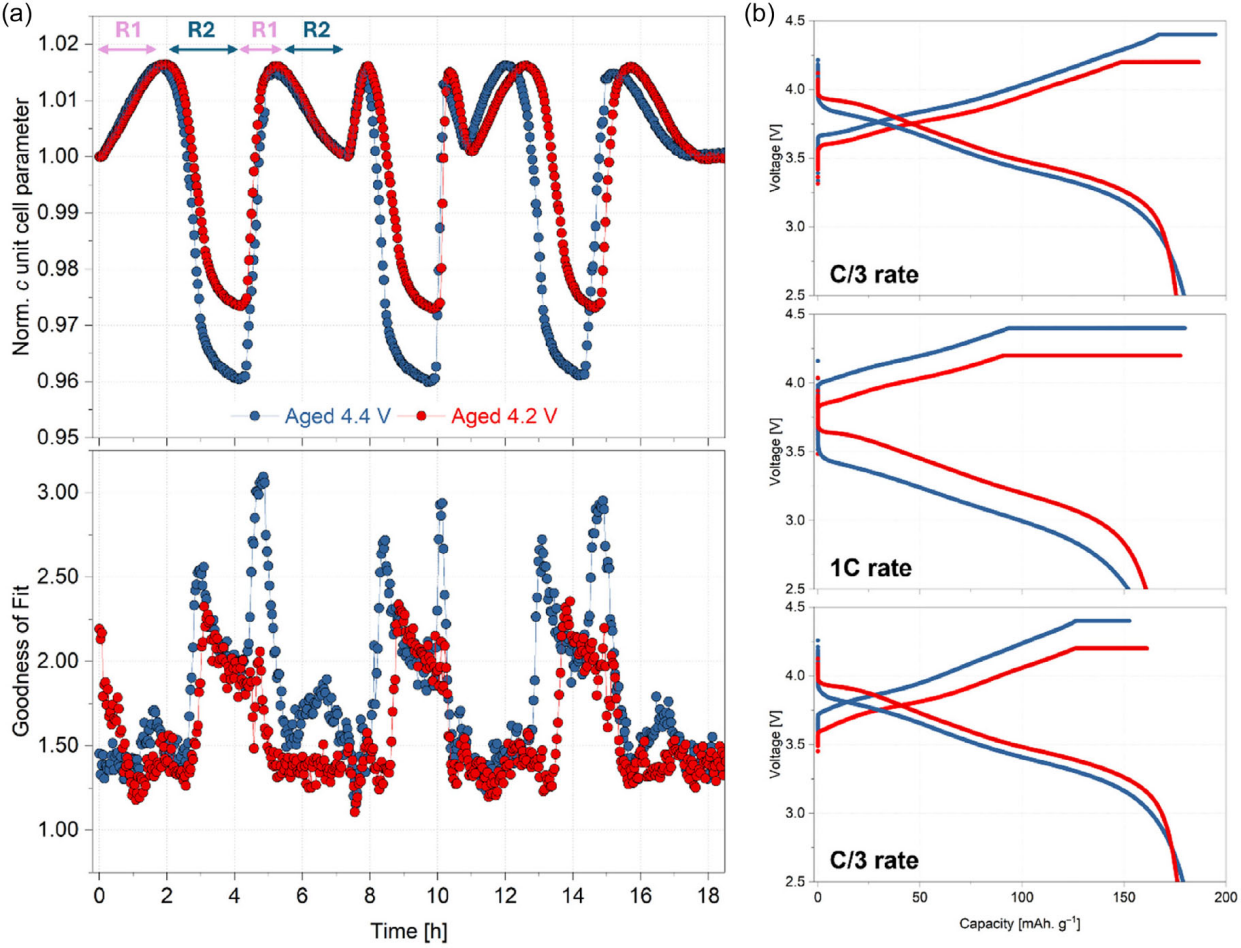
(a) Top: Evolution of the *c*‐lattice parameter normalised to the first value, bottom: corresponding goodness‐of‐fit values obtained from the sequential Pawley analysis of operando XRD data. Labels R1 and R2 in the top panel represent Regime 1 and Regime 2, respectively. (b) Voltage versus capacity profiles from the three C/3, 1C, and C/3 operando cycles.

The NMC811 (003) reflection, and by extension, the *c‐*lattice parameter of the $R\bar{3}$ *m* unit cell, is directly influenced by the state of lithiation of the cathode [30]. This is visible in the sequential Pawley [55] fit results of the operando XRD data, performed using a single phase/unit cell, as shown in Figure 4. Here, the *c‐*lattice parameter exhibits the expected evolution during each of the charge and discharge steps, which can be split into two regimes (note that this is during charge, and is reversed during discharge):

i. Regime 1–From the start of charge to the onset of the *c*‐lattice parameter collapse, where a peak shift towards lower scattering angles is observed. This signifies structural expansion along the layer‐stacking direction and is characterised by an increase in the *c‐*lattice parameter (i.e., increase in interlayer spacing).
ii. Regime 2–From the onset of *c*‐lattice parameter collapse to the end of charge (including the voltage hold), where an abrupt peak shift to higher angles is observed. This signifies structural contraction, characterised by a sharp decrease in the *c‐*lattice parameter (i.e., decrease in interlayer spacing).

As seen in Figure 4a, the extent of *c‐*lattice parameter evolution is larger for the 4.4 V cell. Although this is conventionally used as an indicator of higher (de)lithiation (capacity), the specific charge capacities obtained during the operando cycles are comparable (Figure 4b), similar to that in the standard ageing tests (Figure 1).

This discrepancy between the *c‐*lattice parameter evolution and the capacity arises from the fact that the NMC811 particle bulk segregates into domains with heterogeneous Li^+^ concentrations towards the end of charge. As mentioned previously, this is referred to as fatigued phases, as they show comparatively lesser electrochemical activity than the main (electrochemically active) phase, and manifest in the XRD data as the splitting of the 00*l* reflections as the phases have different interlayer spacings (due to the variations in the Li content) [41]. Pawley fits using a single *R* $\bar{3}$ *m* cell is only able to model the active phase and fails to account for the observed peak splitting of the parent (003) reflection. This is evidenced in the goodness‐of‐fit (GoF) values—the periodic increases in the GoF values, indicative of poor(er) fits, coincide with the end of charge and beginning of discharge, where the highest degree of peak splitting is observed. This intra‐particle concentration gradient arises from the sluggish Li^+^ kinetics within the SC particles, induced by the ageing‐induced surface reconstruction and further compounded by the large particle sizes [13]. Any effect on the bulk delithiation behaviour induced by variations in the surface reconstruction will therefore be directly reflected in how the position, intensity and width of the (003) reflection evolve during cycling. In the following sections, it is demonstrated how changes in the evolution of the NMC811 “(003) peak(s)” reveal heterogeneous bulk delithiation arising from different degrees of particle surface reconstruction.

**FIGURE 5:**
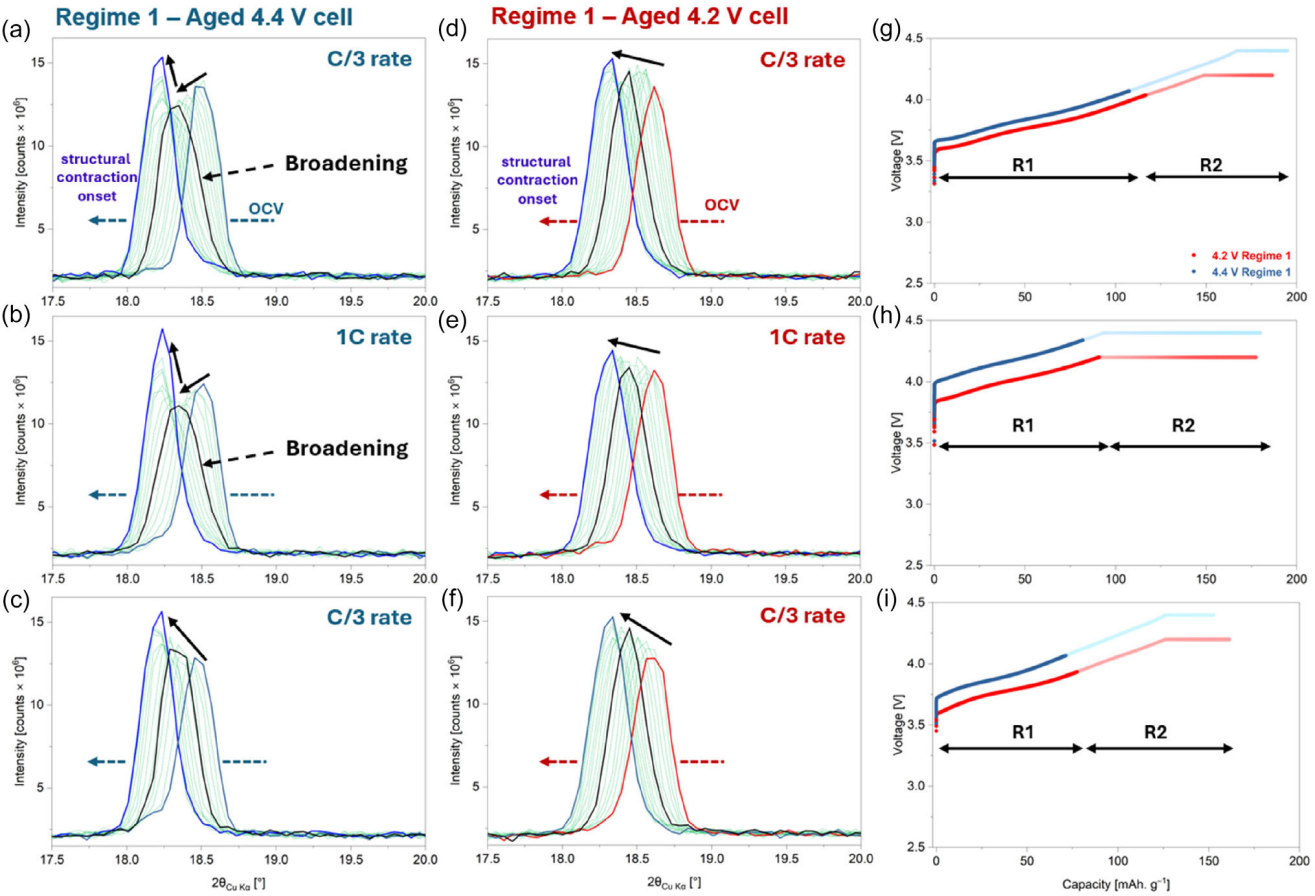
Evolution of the (003) reflection during Regime 1—from the start of charge to the onset of structural contraction for (a–c) the aged 4.4 V cell and (d–f) the aged 4.2 V cell during the operando cycles. (g–i) Corresponding voltage–capacity profiles, where the charge segments associated with Regime 1 are highlighted in darker shades. Regimes 1 and 2 are referred to as R1 and R2, respectively.

Figure 5 shows the evolution of the NMC811 (003) reflection during Regime 1 (start of charge to end of structural expansion/onset of *c*‐lattice parameter collapse) from the 4.4 V (Figure 5a–c) and 4.2 V (Figure 5d–f) cells. For each cell, the three cycles are individually shown for easier comparison. The specific capacities extracted during this regime for each of the cycles are shown in Figure 5g–i. During the first C/3 charge of the aged 4.4 V cell, the (003) reflection demonstrates a notable broadening, characterised by the simultaneous increase and decrease of the peak width and amplitude, respectively (Figure 5a). This broadening indicates the development of heterogeneity in interlayer spacings within the cathode, i.e., the structure segregating into different domains with dissimilar inter‐layer spacings leading to a decrease in the structural correlation along the stacking direction [41]. Upon further charging, the broadening reduces, and the reflection becomes sharper, illustrating an increase in the structural correlation. In the aged 4.2 V cell, where the surface reconstruction is much less severe, no such behaviour is observed in Regime 1. Contrarily, a slight increase in the peak amplitude (with reduction in broadening, Figure 5d) is observed, indicating uniform delithiation and higher structural correlation. The same trends, albeit to a higher degree, are also observed during Regime 1 of the following 1 C cycles in both cells (Figure 5b,e). This is quantified via a single‐peak fitting, as shown in Figure S9. During Regime 1, the total extracted capacity for the 4.4 V cell, are approx. 106 and 94 mAh g^−1^, during the C/3 and 1 C cycles, respectively. This is lower than the aged 4.2 V cell, which shows capacities of approx. 115 and 106 mAh g^−1^, respectively, during the corresponding C/3 and 1 C cycles. This is a direct illustration of how the more severe surface reconstruction in the 4.4 V SC‐NMC811 cathode poses a stronger hindrance to Li^+^ kinetics within the particle from the initial stages of cycling, the effects of which are directly manifested in the evolution of the (003) reflection.

Further corroboration about the influence of the surface reconstruction on the (003) reflection is observed during the final C/3 cycle. Here, the 4.4 and 4.2 V cells reach the onset of *c*‐lattice parameter collapse after extracting approx. 70 and 96 mAh g^−1^, respectively (Figure 5i). Note that the corresponding values from the first C/3 cycle were approx. 106 and 115 mAh g^−1^, respectively. This reduced capacity (for the same C rate) is a direct consequence of the previous faster 1 C cycle, where re‐lithiation of the cathodes occurred to a reduced extent, especially in the 4.4 V cell, due to its more severe surface reconstruction. Consequently, in the following C/3 charge, the cathode operates within a reduced state‐of‐charge window starting from a lower state of lithiation, with the (003) reflection showing no peak broadening unlike the previous two cycles (Figure S9b). The 4.2 V cell shows the same trends as its previous two cycles, further demonstrating how, without major surface reconstruction, the bulk delithiation progresses largely in a homogeneous manner.

**FIGURE 6:**
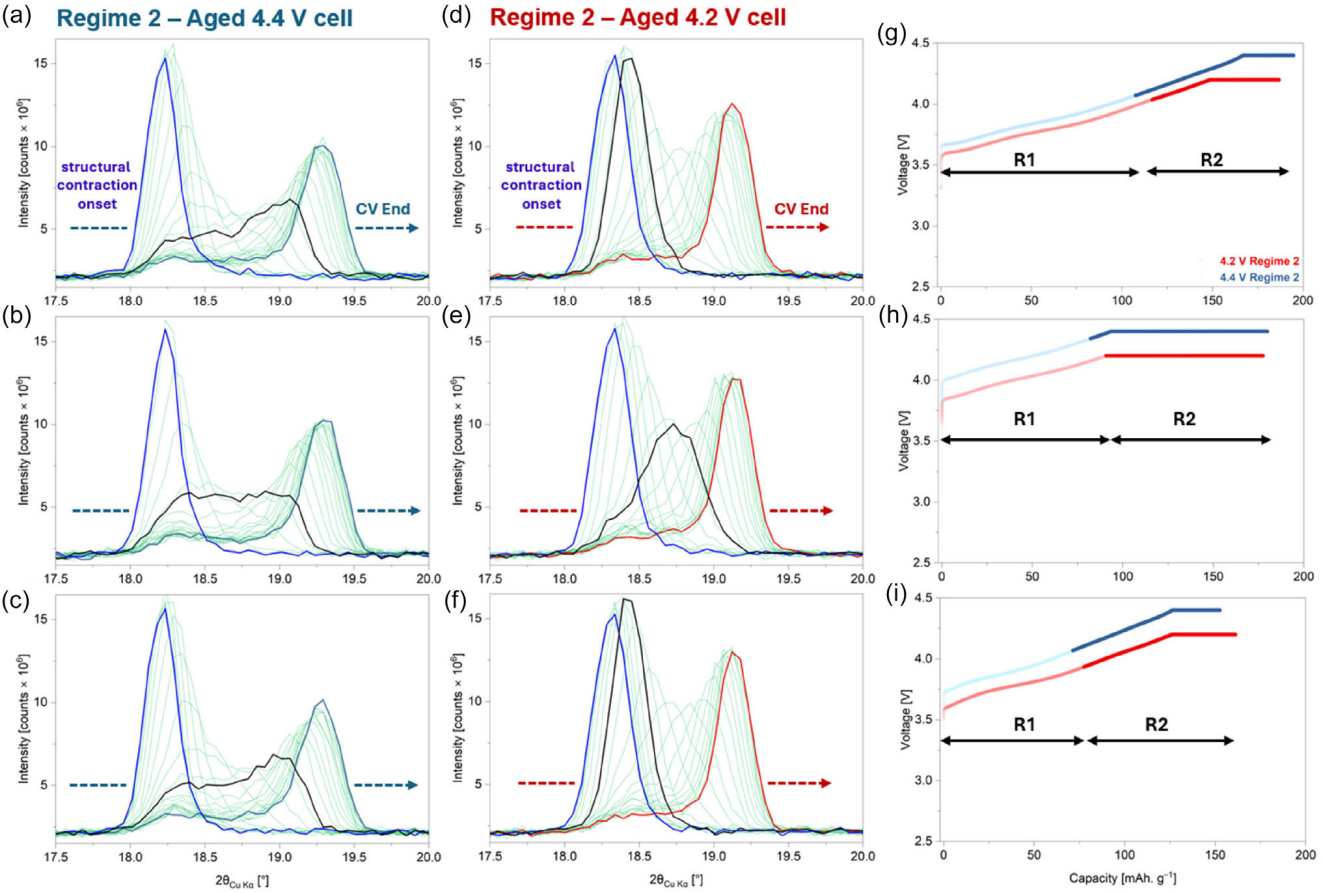
Evolution of the (003) reflection in the Regime 2 (*c*‐lattice parameter collapse) region during charge for (a–c) the 4.4 V cell and (d–f) the 4.2 V cell across the operando cycles. The OCV XRD scans are shown in slate blue (4.4 V cell) and red (4.2 V cell). The black solid line marks the onset of the CV step within Regime 2, except in the aged 4.2 V cell at 1 C, where the blue solid line denotes the CV‐start scan because Regime 2 coincides with the beginning of the CV step. In all other panels, the blue solid line indicates the XRD scan corresponding to the onset of *c*‐lattice parameter collapse. (g–i) Corresponding voltage–capacity profiles, where Regime 2 charge segments are highlighted in darker shades. Regimes 1 and 2 are referred to as R1 and R2, respectively.

Regime 2 extends from the beginning of the *c*‐lattice parameter collapse to the end of the CV hold step, as shown in Figure 6. Here, the 4.4 V cell shows pronounced splitting of the parent (003) reflection into multiple peaks, indicating the separation of the cathode bulk into domains with different Li^+^ concentration (Figure 6a–c). As can be seen, right from the onset of the collapse, there is a substantial decrease in the intensity, with a simultaneous increase in the broadening. Although the angular resolution is insufficient for a reliable deconvolution of the underlying phases, the changes are stark enough to denote the presence of multiple phases. In the aged 4.2 V cell, the domain separation is comparatively less severe and occurs only at a later stage of the collapse, when the cell is already in the CV hold step (Figure 6d,e). As the CV step progresses, the extent of peak splitting reduces significantly in both cells, with the ‘active phase’ being the dominant contributor to the overall diffracted intensity. Notably, comparatively higher fatigue is observed in the 4.4 V cell, as diffuse intensity towards lower scattering angles (18°–19°) from the main (003) reflection. Furthermore, the ratio of the integrated intensity of the diffuse region to that of the main (003) reflection is significantly higher in the 4.4 V cell than in the 4.2 V one (Figure S10), demonstrating higher fatigue in the former. It is important to note that the extracted capacity in Regime 2 during the first C/3 cycle of the 4.4 V cell (87 mAh g^−1^) is substantially higher than that of the 4.2 V cell (70 mAh g^−1^). However, during the following 1 C and C/3 cycles, the difference in extracted capacities progressively diminishes, reaching 96 mAh g^−1^ (4.4 V cell) and 87 mAh g^−1^ (4.2 V cell) at 1 C, and 81 mAh g^−1^ (4.4 V cell) and 84 mAh g^−1^ (4.2 V cell) at C/3 (Figure 6g–i). Despite this progressive reduction in the capacity difference, no corresponding change in the severity of phase separation is observed in either cell, and the overall trends remain consistent across all three cycles. This indicates that, in Regime 2, in addition to surface reconstruction, the structural contraction (i.e., shrinkage of interlayer spacing) plays a dominant role in governing the severity of phase separation. In order to further confirm this observation, the evolution of the (003) reflection in a fresh (after formation) cell (presented in our previous work [13]) is evaluated, where the effect of surface reconstruction is substantially smaller. Corresponding data from the operando XRD experiment on this cell are presented in Figures S11–S13. Here, although no broadening of the (003) reflection is observed during Regime 1 (Figure S12a), pronounced peak broadening is still observed in Regime 2 (Figure S12b). This confirms that structural contraction is primarily responsible for the phase separation in the NMC811 particles. Expectedly, the better Li^+^ kinetics in the absence of any major surface reconstruction in the fresh(er) NMC811 particles also result in the comparatively lower fatigue (Figure S10).

XRD data during discharge from the operando measurements of the aged cells are shown in Figures S14–S16. In Regime 1 of discharge for the 4.4 V cell, where the structure (and *c*‐lattice parameter) expands rapidly, the (003) reflection shows a notable peak splitting (similar to that in Regime 2 of the charge) before coalescing into a singular peak as lithiation proceeds. The same trend is observed in the 4.2 V cathode to a notably smaller extent. At the end of Regime 1 (i.e., at the onset of structural contraction during discharge), the 4.4 V cathode exhibits a comparatively stronger shoulder feature towards higher scattering angles, representative of higher fatigue. This is observed irrespective of cycling rate and demonstrates how a higher degree of surface reconstruction impedes Li^+^ insertion into the particles and promotes inhomogeneous lithiation across the bulk [4]. The 1 C cycle induces the strongest such feature, further underscoring the strong correlation of fatigue to cycling rate kinetics. With continued re‐lithiation during Regime 2 of discharge, the (003) reflection in both cells progressively reverts to states close to those during their previous OCV state. Here, the extent of peak splitting is substantially smaller than in Regime 1, as the expanded structure promotes faster re‐lithiation. Nevertheless, due to the higher degree of surface reconstruction, the evolution of the (003) reflection is markedly non‐uniform in the 4.4 V cell, compared to that of the 4.2 V one. Thus, during discharge, the cathode bulk structure collectively retraces the same structural pathways followed during charge to a large extent. This indicates that the same correlation between surface reconstruction and bulk structural heterogeneity persists during both delithiation and lithiation and can be probed using the multi‐rate operando XRD methodology.

**FIGURE 7:**
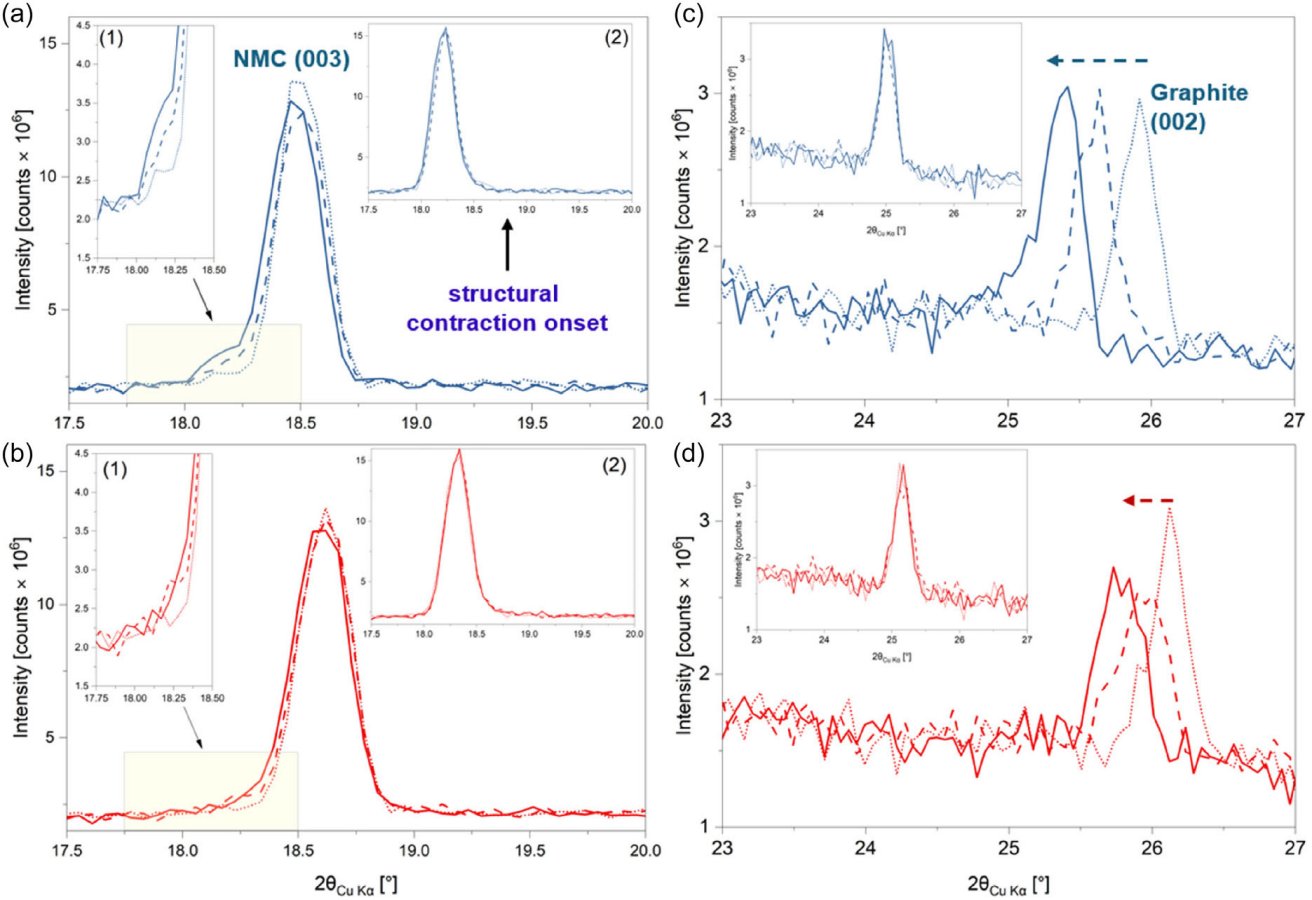
The (003) reflection at the beginning of each operando cycle for the (a) 4.4 and (b) 4.2 V cells. The corresponding graphite (002) reflections from the (c) 4.4 and (d) 4.2 V cells. The two insets—inset 1 and inset 2—in a & b show the enlarged view of the shoulder feature of the (003) reflection at the beginning of each operando cycle and the overlap of (003) reflections at the onset of *c*‐lattice collapse, respectively. The insets in (c & d) represent the graphite peaks at the onset of *c*‐lattice parameter collapse. The short dot, dash, and solid lines denote the data from the first C/3, second 1 C, and third C/3 operando cycles, respectively. The blue and red colours represent the 4.4 and 4.2 V cells, respectively.

It is also important to note that the electrochemical history of the cathode/cell plays a significant role in influencing the (de)lithiation behaviour of the cathode, thereby justifying the need for a multi‐rate operando study (as opposed to a single‐cycle one). This is illustrated in Figure 7, which compares the cathode (003) and graphite (002) reflections at the beginning of each operando cycle for the two cells (i.e., in the OCV state). For the 4.4 V cell, a shoulder feature is visible on the lower‐angle side of the (003) reflection (i.e., the fatigue phase), which becomes progressively more pronounced with cycling, with the final C/3 cycle showing the strongest feature. Due to the higher degree of surface reconstruction in the aged 4.4 V cathode, the faster 1 C cycle leads to a notably lower discharge capacity (Figure S14h) and more inhomogeneous re‐lithiation of the cathode bulk, thereby resulting in the stronger fatigue observed in the subsequent C/3 cycle. The extent of fatigue is comparatively smaller for the 4.2 V cathode because its surface reconstruction is less severe. The increasing trend in fatigue phase formation at OCV has been further quantified using peak fitting of the (003) reflection for both aged cells (Figure S17). Despite the aforementioned differences in the bulk structure, the onset of structural contraction (onset of *c* lattice parameter collapse) still happens at similar states of delithiation in all three cycles (Figure 7a,b, insets 2). These findings are corroborated by the trends observed in the graphite (002) reflections at the corresponding states in the aged cell data (Figure 7c,d). As can be seen, in both cells, the degree of anode lithiation becomes progressively higher with each operando cycle. In other words, the amount of Li^+^ retained in the anode increases with each cycle. The 4.4 V cell expectedly shows a higher degree of anode lithiation in the discharged state for all cycles due to its increased resistance to cathode re‐lithiation due to its more severe surface reconstruction. Similar to the onset of structural contraction, the extent of graphite lithiation at that point is also similar between the three cycles of a cell.

### 2.4 Establishing a Mechanistic Link Between the Surface Reconstruction and Bulk Structure Evolution

**FIGURE 8:**
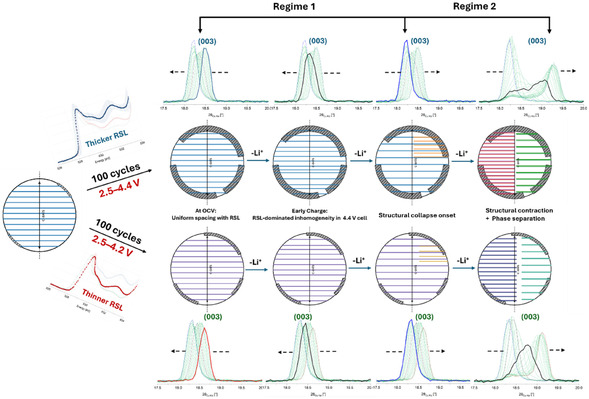
The influence of surface reconstruction on the bulk delithiation behaviour of NMC811 cathode particles. Particles are represented as spheres for clarity. The aged 4.4 V cathode particle has a thicker, inward‐grown reconstructed surface layer (RSL, grey shaded region) with greater coverage than its 4.2 V counterpart, while the fresh particle has minimal surface reconstruction. The NMC unit cell *c*‐lattice parameter passes through the particle centre, with the (003) planes stacked along this direction, and Li^+^ diffusion occurs along the (003) planes, perpendicular to the stacking direction. The evolution of interlayer spacing during delithiation is represented using different line colours: uniform interlayer spacing at OCV (blue/purple), the development of local inhomogeneity at early charge (mixed blue/orange), the onset of *c*‐lattice parameter collapse (orange), and pronounced structural contraction (along (003)) with phase separation at high states of charge (co‐existing red and green phases). Increasing hindrance to delithiation imposed by the surface reconstruction layer leads to spatial heterogeneity in interlayer spacing, which manifests as progressive peak broadening, asymmetry, and eventual splitting of the parent (003) reflection into multiple peaks.

The primary Li^+^ diffusion pathway in Li*TM*O_2_‐type layered oxide cathodes such as NMC811 is within the basal (ab) planes, whose interlayer spacing are represented by the position of the (003) reflection. Any changes to the Li^+^ transport during cycling along this plane directly manifest in the evolution of the position, width, and integral intensity of this reflection. The results from this work, highlighting the mechanistic interplay between surface reconstruction and bulk structural delithiation, are schematically visualised in Figure 8.

In beginning of charge (Regime 1), the structural expansion along the stacking direction favours Li^+^ extraction. However, the reconstructed surface layer locally impedes Li^+^ transport, counteracting the effects of expansion and creating Li^+^ concentration gradients within the particles. This leads to segregation of the particle bulk into distinct inhomogeneous domains with different interlayer spacings, leading to a reduction in the structural coherence along the stacking direction. In the aged 4.4 V cathode with a higher degree of surface reconstruction, the (003) reflection initially broadens during early stages of charging, which reflects inhomogeneity in interlayer spacing caused by the influence of the reconstructed surface layer. As delithiation progresses in this regime, the structural expansion begins to outweigh the negative effects of surface reconstruction on Li^+^ extraction, leading to a reduction in the broadening of the (003) reflection until the onset of the structural contraction, marked by the *c*‐lattice parameter collapse. This corresponds to an increase in the structural coherence along the stacking direction. Despite the visible recovery in the crystallographic ordering, the cathode bulk still possesses domains that lag in terms of delithiation. These domains have a higher Li^+^ content and narrower interlayer spacings, which is evident in the XRD data as a small shoulder feature in the (003) reflection towards higher scattering angles. In Regime 2, both structural contraction and reconstructed surface layer suppresses Li^+^ extraction; however, this does not imply that their effects are non‐competing. Due to spatial differences in the extent of surface reconstruction, domains/regions will experience different degrees of resistance to delithiation. This spatial variance in the Li^+^ mobility leads to pronounced domain segregation towards the end of charging, where Li^+^ diffusion out of the particles is blocked both at the surface (by the surface reconstruction) and in the bulk (by the contracted phases). This mechanistic view of Li^+^ ‘trapping’ in the aged 4.4 V cathode is evident by the formation of a broad secondary feature in the (003) reflection towards lower scattering angles, illustrating the convolution of multiple phases with different Li^+^ concentration. In the aged 4.2 V cathode, the same competing mechanisms operate. However, due to the lesser surface reconstruction, structural expansion in Regime 1 dominates and consequently, no such broadening and decrease in the intensity of (003) reflection is observed until the onset of structural contraction. In Regime 2, a diffuse secondary feature in the (003) reflection is formed, similar to the 4.4 V cathode, albeit with comparatively lower intensity. The major distinction between the aged 4.4 and 4.2 V cathodes in Regime 2 lies in the severity of phase separation and the corresponding extent of fatigue phase formation. In the aged 4.4 V cathode, the more pronounced phase separation manifests as a clear splitting of the (003) reflection at the intermediate state, whereas in the aged 4.2 V cathode, the phase separation is comparatively milder and appears primarily as broadening of the (003) reflection rather than full splitting. Consequently, the extent of fatigue phase formation is higher in the former and lower in the latter.

## 3 Conclusion & Outlook

This work demonstrates how the layered‐to‐spinel/rocksalt surface reconstruction in SC Ni‐rich layered oxide cathodes strongly modulates bulk delithiation behaviour and the associated structural heterogeneity upon electrochemical ageing. Different degrees of surface reconstruction were induced in SC NMC811 cathode particles by ageing them in single‐layer pouch full cells (vs. graphite) within two voltage windows, 2.5–4.2 and 2.5–4.4 V, for 100 cycles at C/3 rate and 40°C. Because it exceeds the oxygen‐evolution threshold (∼4.3 V vs. Li^+^/Li), the 4.4 V cathode exhibited a higher degree of surface reduction, with no notable changes in the sub‐surface or bulk. No particle cracking was observed in either cathode. The higher degree of surface reconstruction in the 4.4 V cathode more strongly impeded Li^+^ transport across the cathode–electrolyte interface, resulting in a larger rise in cell resistance and faster capacity fade. Tracking the real‐time evolution of the NMC811 (003) reflection using a multi‐rate operando XRD methodology (C/3–1C–C/3) showed that increased surface reconstruction reduces Li^+^ mobility and structural coherence along the layer‐stacking direction during structural expansion, and promotes more severe phase separation during the ensuing structural contraction. Structural contraction emerges as the intrinsic driver of phase separation, while the extent of bulk heterogeneity and fatigue is strongly modulated by the degree of surface reconstruction. This behaviour becomes more severe over the course of cycling, highlighting how the kinetics‐driven capacity becomes more severe with prolonged cycling.

Overall, these results demonstrate that nanometric surface‐level changes can exert a disproportionate influence on Li^+^ transport kinetics in SC Ni‐rich layered oxide cathodes, particularly after long‐term electrochemical ageing. Understanding how Li^+^ transport evolves across crystallographic, particle, and electrode length scales will be key to devising effective mitigation strategies, whose impact must be validated at the cell level under representative conditions. In this context, non‐destructive, pouch‐cell‐compatible diagnostics such as the approach presented here offer a practical route to quantify surface–bulk coupling and parameterise kinetics‐driven bulk transformations. Understanding how these properties evolve in greater detail over extended cycling, across a wider range of operating conditions, will help guide the development of improved SC Ni‐rich layered oxide Li‐ion battery cathodes.

## 4 Experimental Section

### 4.1 Fabrication and Assembly of Single‐Layer Pouch Cells

The single‐layer pouch cells were fabricated following established standard operating procedures on the WMG battery scale‐up pilot line (TRL‐5) in a dry‐room environment (dew point −45°C), similar to our earlier works [4, 30]. In brief, the cathode slurry, composed of Li_1.01_Ni_0.81_Mn_0.06_Co_0.11_O_2_ (NMC811, 95.5 wt.%), PVDF (2.5 wt.%), and conductive carbon (2 wt.%), was mixed using a Bühler high‐torque mixer and coated onto a 15‐μm aluminium foil to a target coating thickness of ∼45 μm. The anode slurry, containing graphite, CMC, SBR, and conductive carbon in a weight ratio of 95.5:1.5:2.25:1, was coated onto 10.2‐μm copper foil. The average areal coat weights were ∼12.3 mg cm^−2^ (cathode) and ∼10.8 mg cm^−2^ (anode). The electrodes were calendared to press densities of 2.7 g cm^−3^ (cathode) and 1.4 g cm^−3^ (anode), and subsequently cut to desired dimensions, yielding electrode areas of 33.22 cm^2^ (cathode) and 35 cm^2^ (anode). Aluminium (for cathode) and Ni‐coated Cu (for anode) current‐collector tabs were ultrasonically welded, and the electrodes were stacked in a cathode–separator–anode configuration using a Celgard 2325 trilayer microporous membrane (25 μm). The stacks were packaged in aluminium‐laminated pouches, filled with 1 g of LP57 electrolyte (1 M LiPF_6_ in EC:EMC = 3:7 vol%) containing 2 wt.% vinylene carbonate, and heat‐sealed under vacuum. The approx. areal capacity of the cells was ∼2.1 mAh cm^−2^.

### 4.2 Electrochemical Testing

After assembly, the pouch cells were cycled using a Maccor 4000 series cycler. The cells were first held at 1.5 V for 20 h at 40°C in a climate chamber to ensure uniform electrolyte wetting. Subsequently, two formation cycles were carried out under galvanostatic conditions at ∼C/20 (1 C = 200 mAh g^−1^) within two voltage windows –2.5–4.2 and 2.5–4.4 V. No degassing step was performed, as gas evolution during formation was negligible. Following formation, both cell types were aged using a CCCV protocol during charge and a CC protocol during discharge at C/3 and 40°C, within their respective voltage windows (2.5–4.2 and 2.5–4.4 V) for 100 cycles. The CV hold at the upper cutoff voltage was terminated when the current decreased to C/30. Potentiostatic EIS was performed after formation and after 100 cycles using a BioLogic VMP3. Prior to measurement, cells were CC charged to 3.8 V at C/20. Then, EIS spectra were collected at 3.8 V and 25°C with a 10 mV perturbation over the frequency range 100 kHz to 10 mHz. The impedance spectra were analysed using the impedance.py fitting package [56]. The selected ECM consisted of a series resistance (*R*_0_), followed by three parallel interfacial resistance elements (*R*_1_–*R*_3_), each paired with a constant phase element (CPE_1_–CPE_3_), together with an additional low‐frequency constant phase element (CPE_4_). These elements are typically associated with the ohmic resistance, surface/SEI contribution, anode charge–transfer process, cathode charge–transfer process, and solid‐state diffusion behaviour, respectively [36]. Diagnostic C/10 cycles with ICI steps were also performed after formation and after ageing. Here, the current was paused every 10 min for 5 min during charge and discharge. No interruptions were applied during the CV hold at the top of charge (current terminated to C/30). Internal resistance was determined at each pause using a Python‐based fitting routine by fitting the voltage drop (Δ*V*) versus √time with linear regression, extrapolating the fit back to time, *t* = 0 to obtain the instantaneous voltage drop, and dividing this value by the applied current (*I*) [46, 47, 48]. A covariance matrix was calculated for the purpose of error propagation, and the fitting parameters were iteratively refined until a satisfactory fit was achieved. An additional diagnostic cycle comprising CCCV charge and CC discharge at C/10 was performed after formation and after the ageing for both cells to check the capacities.

### 4.3 Operando X‐Ray Diffraction and Data Analysis

Operando X‐ray diffraction measurements were performed at room temperature using a BioLogic SP150 potentiostat. The cells were cycled within their respective voltage windows of 2.5–4.2 and 2.5–4.4 V. Three consecutive operando cycles were conducted at C/3, 1 C, and C/3 using a CCCV protocol during charge and a CC protocol during discharge. In each cycle, the CV step was terminated at a lower cut‐off current of C/30, followed by a 10 min rest under open‐circuit conditions. X‐ray diffraction data were collected directly from the pouch cells using a Xenocs Xeuss 2.0 system with Cu Kα radiation (*λ* = 1.5418 Å). Data were continuously acquired with an exposure time of 120 s per scan using a detector with a pixel size of 0.172 mm × 0.172 mm. Additionally, 300 s diffraction scans were collected immediately prior to the start of the operando experiment for both the 4.2 and 4.4 V cells. The sample to detector distance was calibrated prior to the measurements using a LaB_6_ standard, and appropriate geometrical corrections were applied to account for detector alignment and sample orientation. Two‐dimensional diffraction images were converted into one‐dimensional patterns by azimuthal integration using the Xenocs XSACT software. The operando XRD data were analysed using TOPAS Academic (version 7) [57]. Sequential Pawley analysis was carried out using NMC811 and aluminium as the two contributing crystalline phases, with the graphite phase excluded from the fitting. The NMC811 phase was modelled using a rhombohedral unit cell (*R* $\bar{3}$ *m* space group symmetry) with initial unit cell parameters of *a* = 2.87 Å and *c* = 14.2 Å, whereas the Al phase was described using a cubic unit cell (space group *Fm* $\bar{3}$ *m*) with an initial lattice parameter of *a* = 4.04 Å. The background was modelled using a fourth degree polynomial function, and peak shapes were described using the Thompson–Cox–Hastings (TCH) pseudo‐Voigt function, as defined in the Topas programme. The refinement was limited to the (003) and (101) reflections of the NMC811 phase and the (111) reflection of Al. The quality of the fits was evaluated using the R‐weighted pattern (*R*_wp_) and goodness of fit (GoF) parameters (definitions are found in the Topas technical reference manual). The operando XRD data for the fresh SC‐NMC811 cathode included in this study were taken from our earlier work [13]. Single‐peak fitting of the (003) reflection using the TCH pseudo‐Voigt function to extract the normalised integral intensity and full width at half maximum (FWHM) was also performed in TOPAS Academic (version 7). The fitting of the fatigue phase of the (003) reflection was performed using Origin software, employing a Gaussian peak‐shape function. The number of fatigue peaks included in the fitting was determined based on the spatial extent of the diffuse intensity region of the (003) reflection in order to achieve an optimal fit.

### 4.4 Soft X‐Ray Absorption Spectroscopy and High‐Resolution Resonant Inelastic X‐Ray Scattering

The 4.4 and 4.2 V cathode samples for measurement were extracted by disassembling the fully‐discharged pouch cells (to 2.5 V) in an argon‐filled glovebox. The extracted electrodes were cut to an appropriate size, rinsed with dimethyl carbonate, and dried thoroughly before being loaded onto sample pucks, which were then transferred to the spectrometer under vacuum.

The X‐ray absorption spectroscopy and RIXS measurements were performed at the I21 beamline at the Diamond Light Source (Didcot, UK) [45]. All measurements were carried out in ultra‐high vacuum conditions at 40 K. Soft XAS data at the O K‐ (520–580 eV), Mn L‐ (630–660 eV), Co L‐ (770–810 eV) and Ni L‐edges (845–885 eV) were continuously collected in the TEY and TFY modes using the standard beamline setup. The former was measured based on the drain current from the sample, and the latter using two photodiodes located at different locations with respect to the samples. In all cases, the measured spectra were normalised to the incident flux before analysis. The O K‐edge data were processed using Athena software, [58] similar to the conventional practice for TM K‐edge data. Background subtraction of the Ni, Co, and Mn L‐edge data was carried out using Origin software. All normalisations were also performed using Origin. The O, Mn, Co, and Ni data were normalised to the peak/feature at approx. 529, 644, 782, and 858 eV, respectively. For the Mn L‐edge, the raw absorption data was inverted with uncharacteristically poor signal‐to‐noise ratio, presumably due to self‐absorption and experimental geometry effects [59, 60]. Therefore, the data was inverted and smoothed for comparison.

O K‐edge RIXS line scans were collected on different sample locations using excitation energies of 531 and 531.2 eV. Each scan was collected for 120 s. For each RIXS spectrum, carbon tape was measured as a reference to calibrate the position of the elastic peak (0 eV). The energy resolution was approx. 0.03 eV. The raw datasets were processed using the DAWN software package in accordance with standard beamline data‐reduction protocols [61].

Corresponding data from the pristine NMC811 cathode from our previous work [4] was used purely for comparative purposes in the Supporting Information.

### 4.5 Cross‐Sectional Scanning Electron Microscopy (SEM)

Cathode cross sections for SEM were prepared by Ar broad‐beam ion milling. Samples were prepared in an Ar glove box (similar to the X‐ray spectroscopy ones) and then transferred to an Hitachi IM4000Plus ion‐milling system. The cross‐sections were then transferred without air exposure to a Thermo Fisher Scientific Scios Dualbeam using the CleanConnect inert gas transfer system for imaging [62]. Images were acquired using accelerating voltages of 2 or 3 kV and current of 100 pA.

## Supporting Information

Additional supporting information can be found online in the Supporting Information section.

## Funding

This study was supported by Faraday Institution (FIRG065, FIRG062, FIRG060, FIVRF005), and the Hartnoll Centre for Experimental Fuel Technologies.

## Conflicts of Interest

The authors declare no conflicts of interest.

## Supporting information

Supplementary Material

## Data Availability

The data that supports the findings of this study are available in the supplementary material of this article.
