# Supplementary material for: How Surface Reconstruction Drives Heterogeneous Bulk Delithiation in Single‐Crystalline Ni‐Rich Layered Oxide Cathodes
Source: Small Sci. 2026 May 12;6(5):e70286. doi: 10.1002/smsc.70286 (PMC13165927; doi:10.1002/smsc.70286)
Supplement: Supplementary file 1 — Supplementary Material [file SMSC-6-e70286-s001.pdf]

Supplementary Information File

How Surface Reconstruction Drives Heterogeneous Bulk Delithiation in Single-crystalline Ni-rich Layered Oxide Cathodes

Gaurav C. Pandey,[a] Ashok S. Menon,[a,b*] Valeria Calani San Miguel,[a,b] José J. Arroyo-Gómez,[a,b,c,d] Harry Gillions,[a] Rebecca Sellers,[a,b] Matthew J. W. Ogley,[a,b] Eleni Fiamegkou,[a,b] Satish Bolloju,[a] Sahil Tippireddy,[e] Mirian Garcia-Fernandez,[e] Steven Huband,[f] Louis F. J. Piper[a,b*]

[a] Warwick Manufacturing Group (WMG), University of Warwick, Coventry, CV4 7AL, UK
[b] The Faraday Institution, Quad One, Harwell Science and Innovation Campus, Didcot, UK
[c] Departamento de Almacenamiento de la Energía, Subgerencia Operativa de Energía y Movilidad, Instituto Nacional de Tecnología Industrial (INTI), Avenida General Paz 5445, San Martín, Buenos Aires 1650, Argentina
[d] Consejo Nacional de Investigaciones Científicas y Técnicas (CONICET), Godoy Cruz 2290, Argentina
[e] Diamond Light Source Ltd., Harwell Science and Innovation Campus, Didcot, OX11 0DE, UK
[f] Department of Physics, University of Warwick, Coventry CV4 7AL, UK

*Corresponding authors' emails: ashok.menon@warwick.ac.uk, louis.piper@warwick.ac.uk

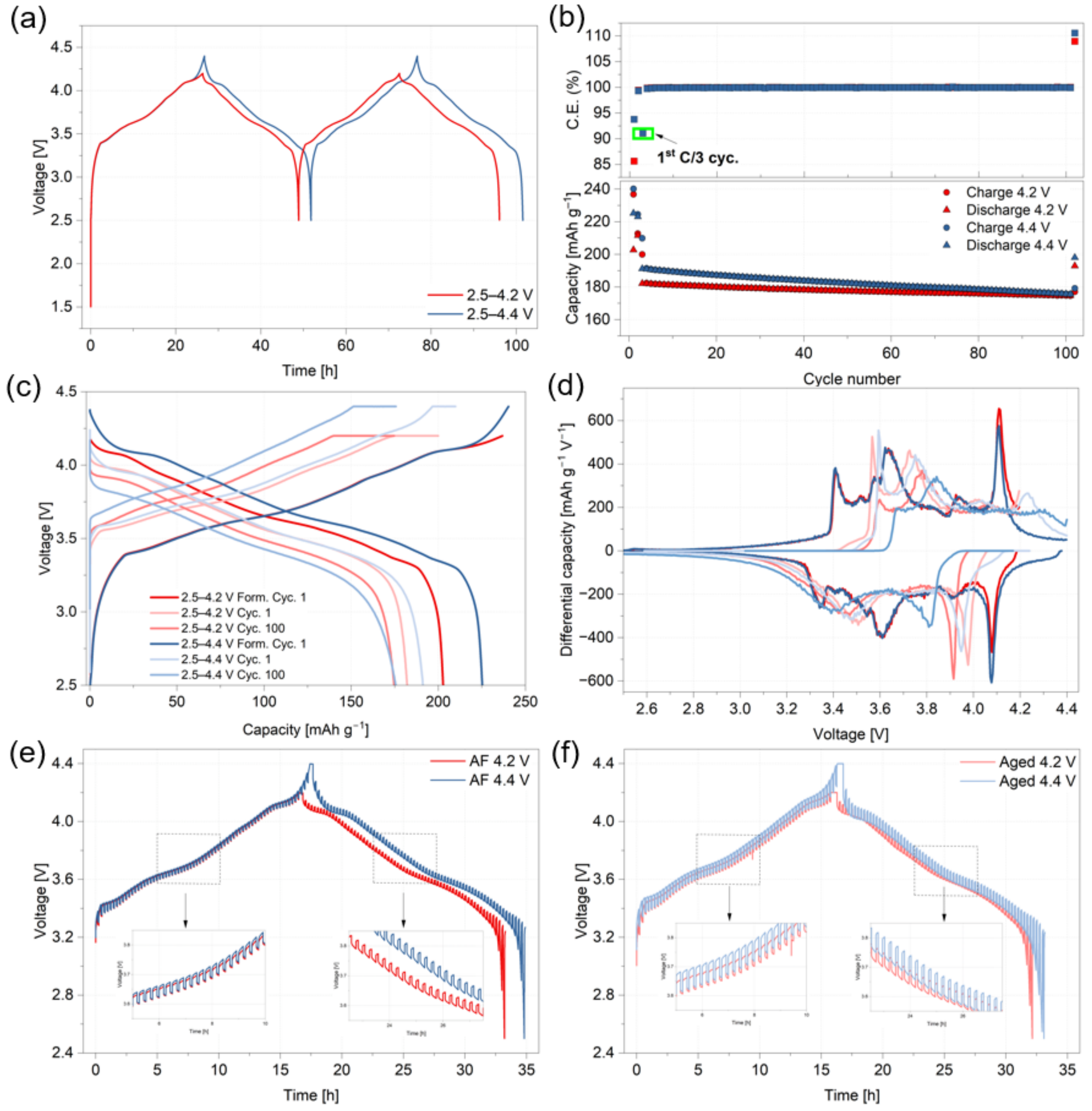

Figure S1: *(a) Voltage–time profiles of the two C/20 formation cycles performed on the single-layer pouch cells cycled within 2.5–4.2 V and 2.5–4.4 V at 40 °C. (b) Specific charge–discharge capacities and Coulombic efficiencies from the C/20 formation cycles, the C/3 aging, and the final C/10 diagnostic cycle. (c) Voltage–capacity and (d) differential-capacity profiles from the first formation cycle and the first and last C/3 cycles of the aging test. (e) Voltage–time profiles collected during ICI-diagnostic cycling after formation and after aging, with the voltage drops/spikes during charge/discharge shown in the insets.*

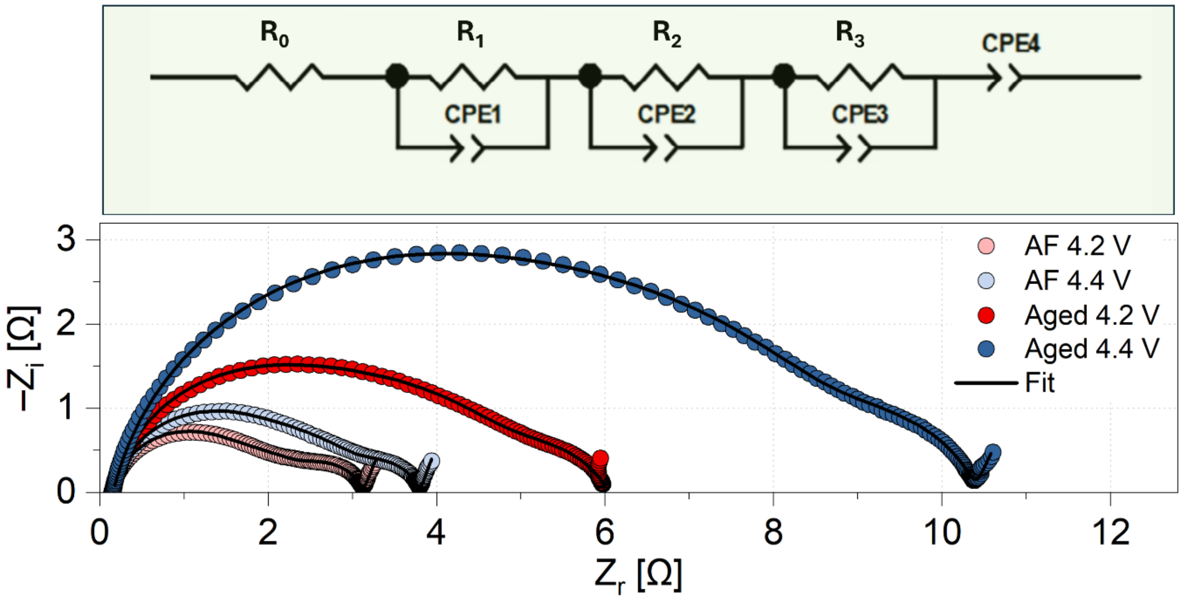

Figure S2: *The equivalent circuit model used for fitting the electrochemical impedance spectroscopy data (top), The corresponding Nyquist plots (bottom) show the experimental data and fitted curves for the two cells measured at 3.8 V, after formation (AF) and aging. R and CPE in the fitting model represent resistors and constant phase elements, respectively.* Z_r *and* Z_i *denote the real and imaginary impedances, respectively.*

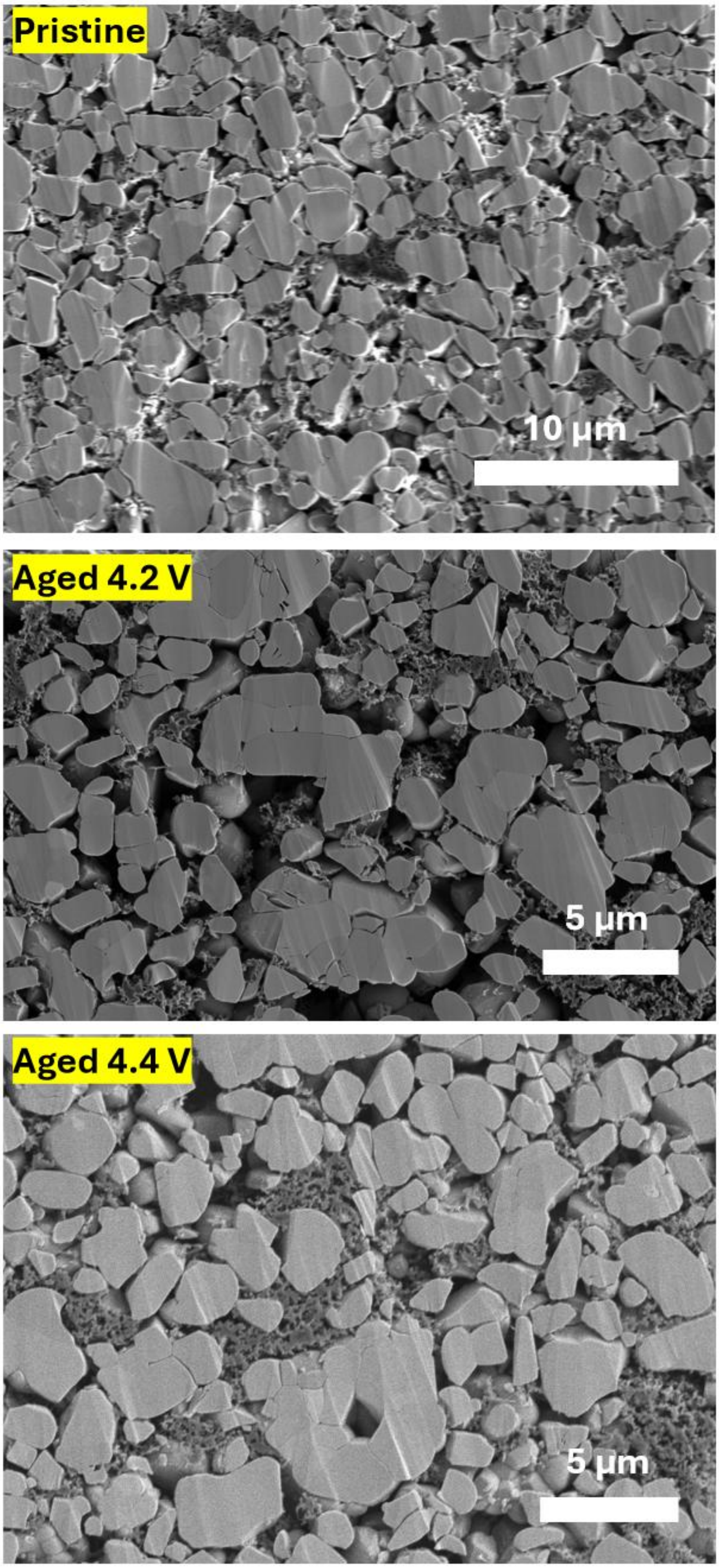

Figure S3: *Cross-sectional SEM images of pristine, aged 4.4 V and aged 4.2 V NMC811 cathodes.*

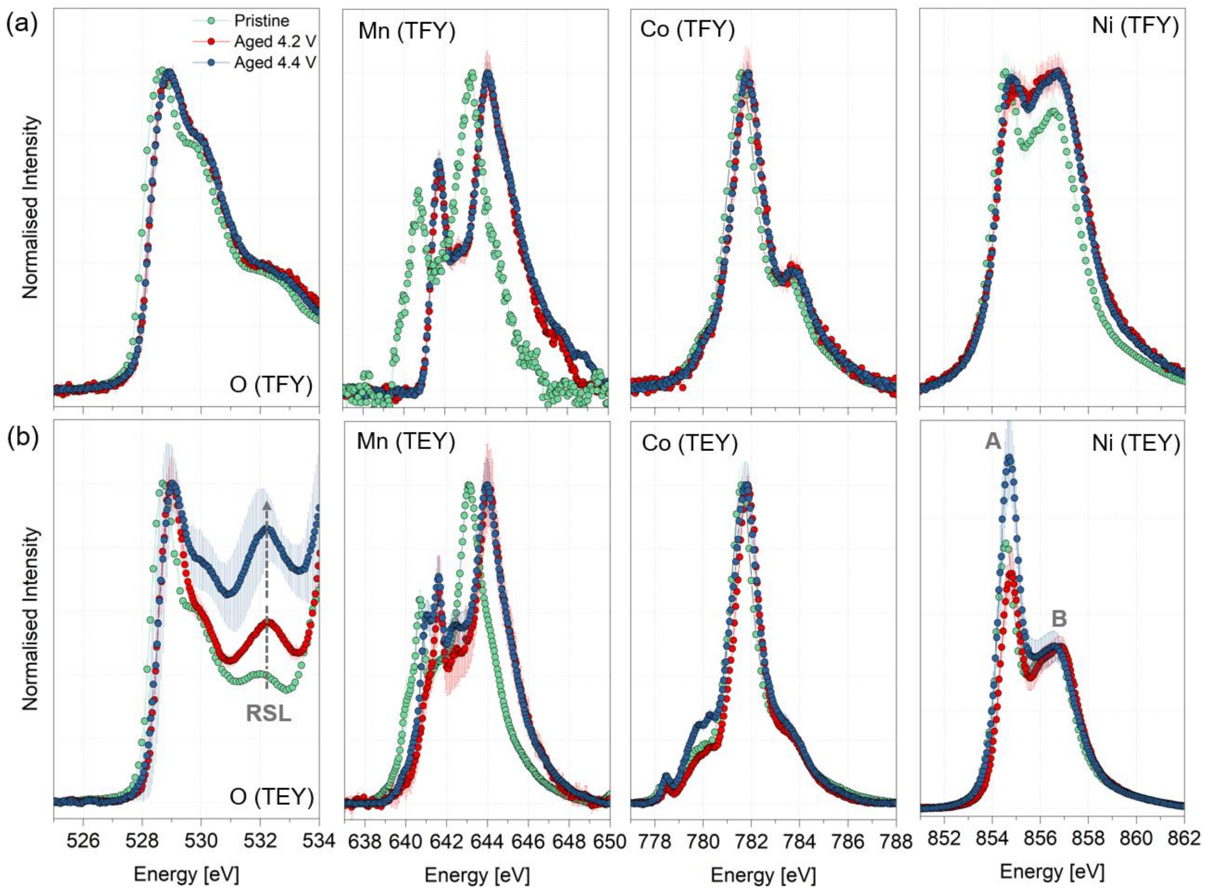

Figure S4: *Background-subtracted and normalised soft XAS spectra of the aged 4.2 V and 4.4 V cathodes in the discharged state, with a pristine SC-NMC811 cathode as reference measured for our previous work* [1]. *(a) O K-edge and Mn, Co, and Ni* $L_{2,3}$*-edge spectra collected in total fluorescence yield (TFY) mode. (b) Corresponding O K-edge and transition-metal* $L_{2,3}$*-edge spectra collected in total electron yield (TEY) mode. The apparent rigid energy offset observed in the pristine SC-NMC811 dataset arises because it was acquired during an earlier beamtime session under slightly different experimental conditions, and a consistent post-measurement energy calibration was not performed. The absolute energy scales are not directly comparable between the pristine and aged cathode datasets. The comparison presented here is intended to be strictly qualitative rather than quantitative.*

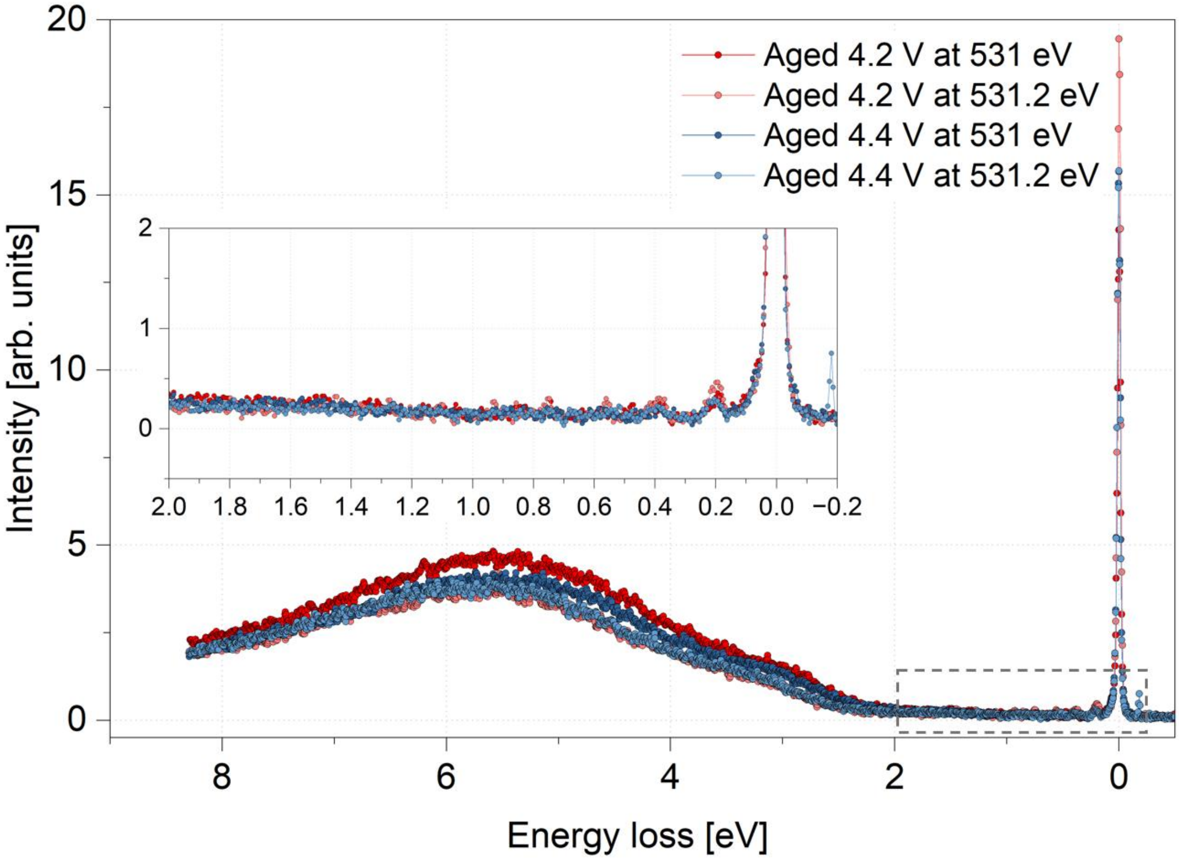

Figure S5: *O K-edge RIXS spectra of aged 4.2 V and 4.4 V cathodes collected with incident X-ray energies of 531.0 eV and 531.2 eV. The region close to the elastic peak is highlighted in the inset.*

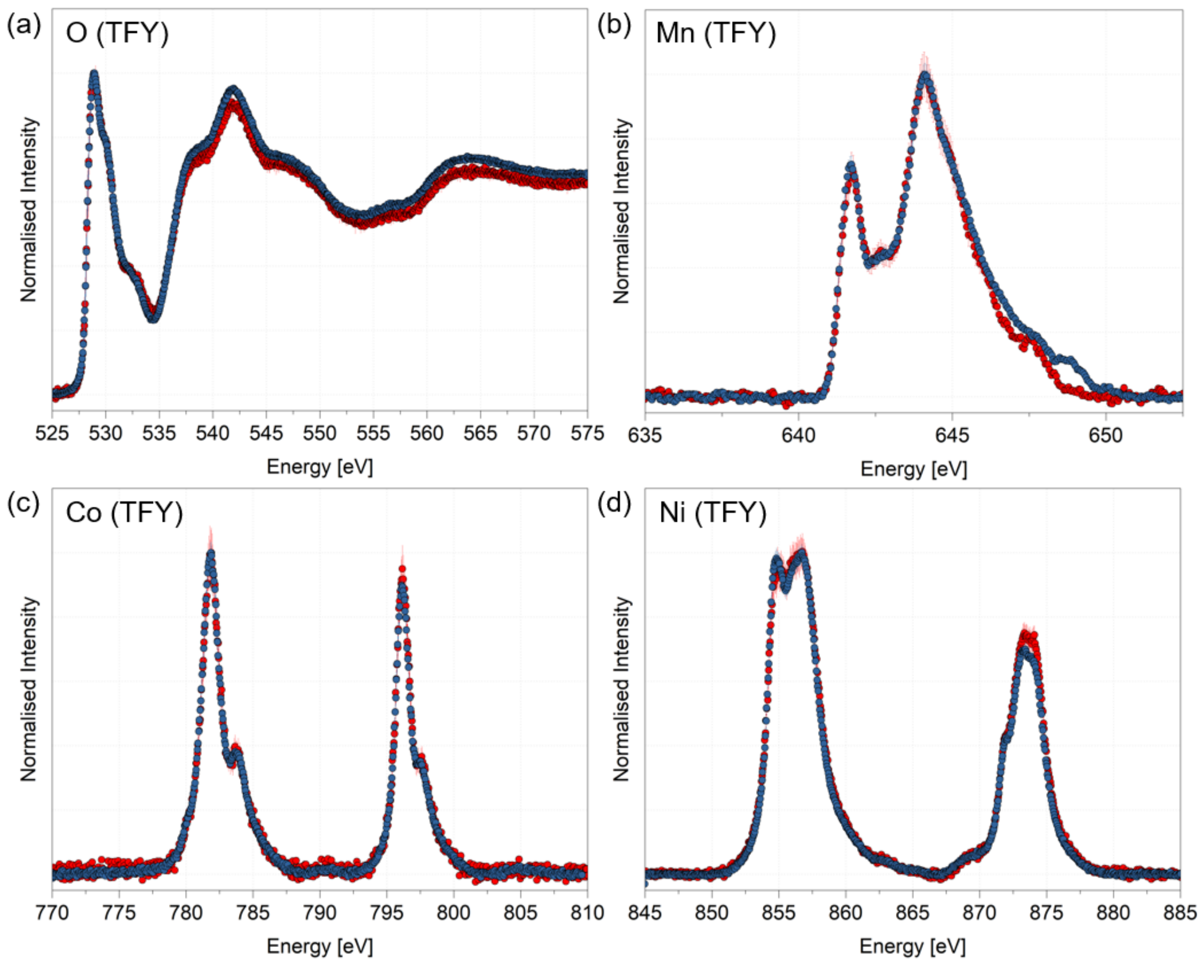

Figure S6*: The full (a) O K-, (b) Mn $L_{2,3}$- (c) Co $L_{2,3}$-, and (d) Ni $L_{2,3}$-edge spectra collected in total fluorescence yield (TFY) mode. The Mn L_2-edge is not shown due to the poor signal to noise ratio above 650 eV arising from self-absorption and experimental geometry effects.*

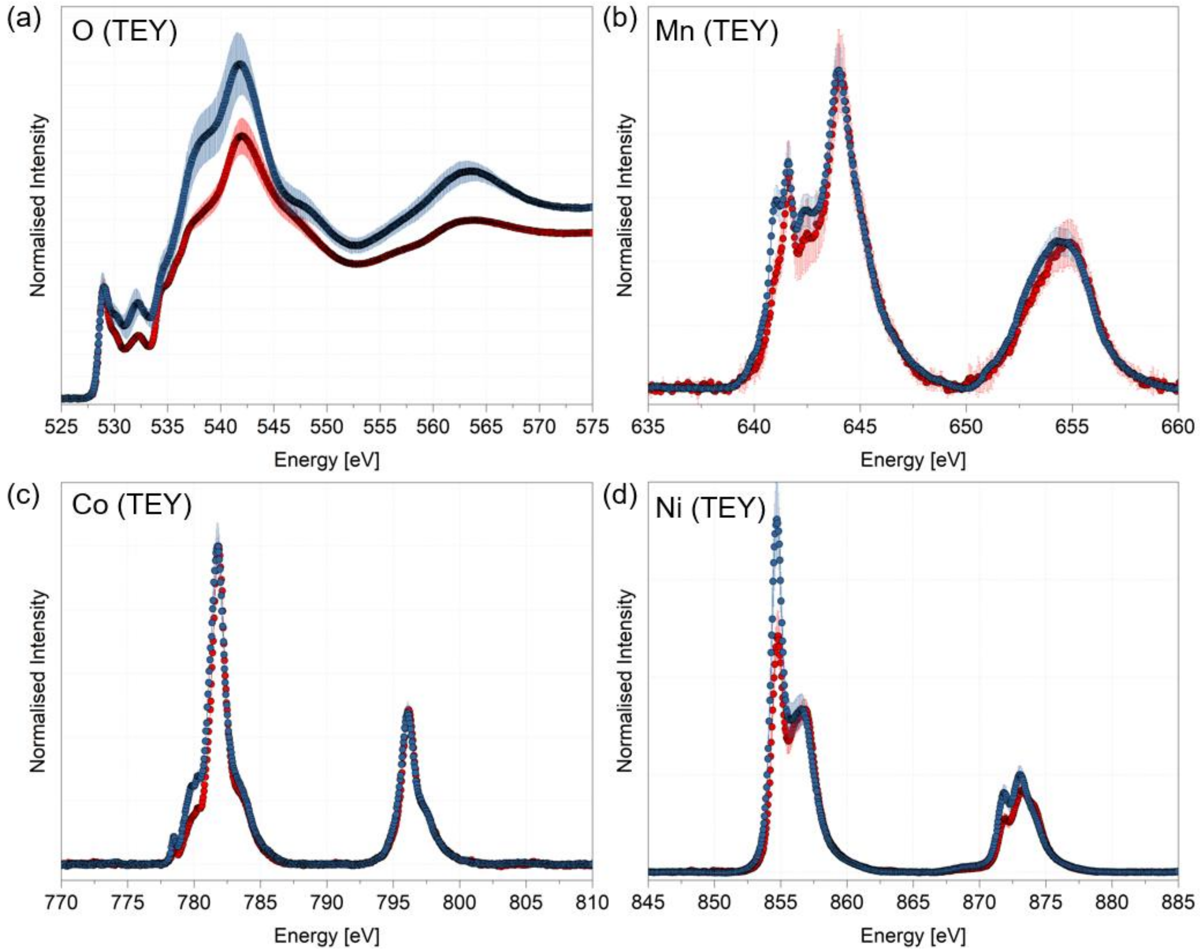

Figure S7*: The full (a) O K-, (b) Mn $L_{2,3}$- (c) Co $L_{2,3}$-, and (d) Ni $L_{2,3}$-edge spectra collected in the total electron yield (TFY) mode.*

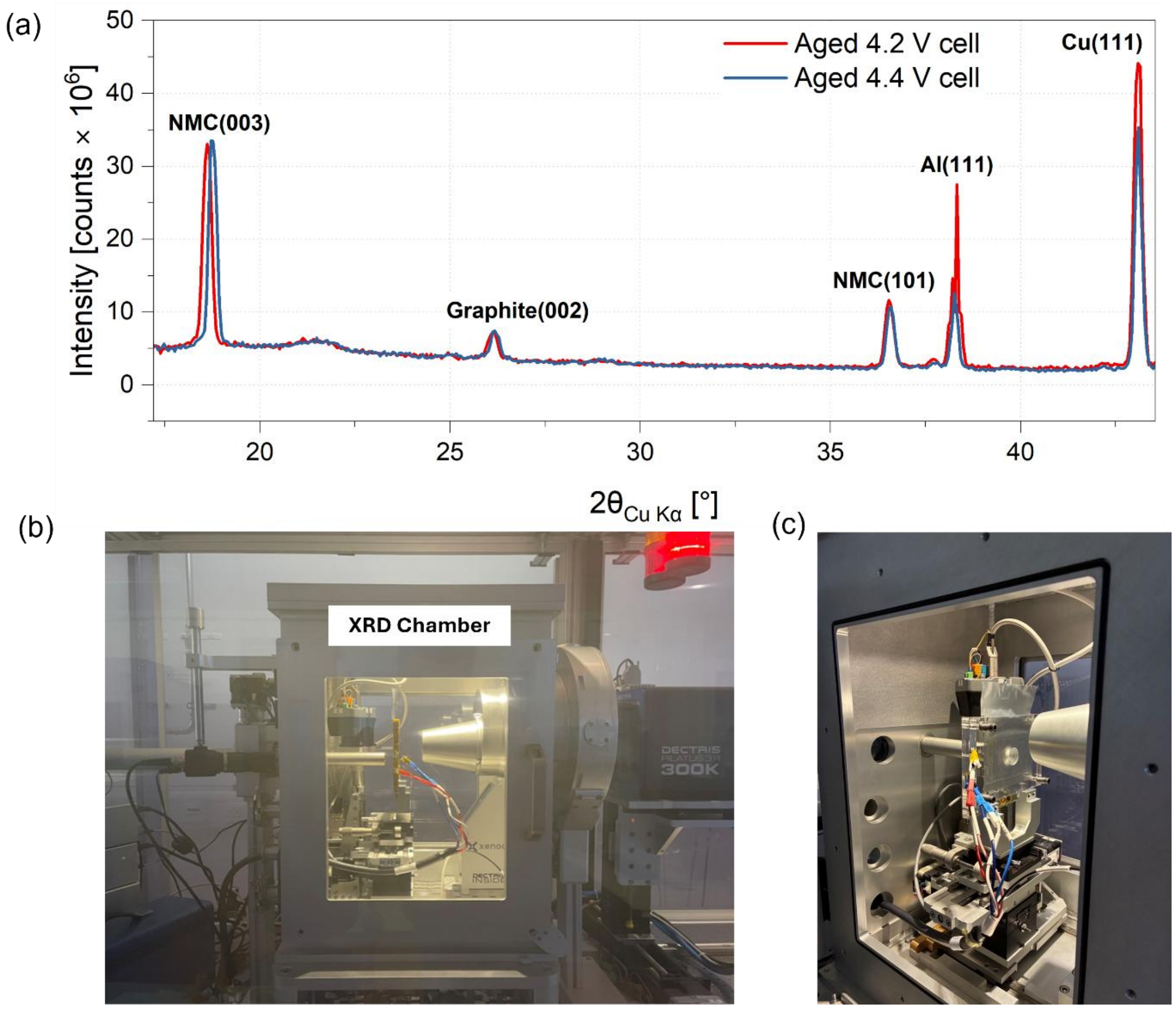

Figure S8: *(a) Five-minute XRD scans collected at open-circuit voltage (OCV) for the aged 4.2 V and 4.4 V cells immediately prior to the operando experiments. (b)The Xenocs Xeuss 2.0 instrument used for the multi-rate operando XRD measurements of single-layer pouch cells. (c) X-ray diffractometer chamber showing the experimental configuration used during operando measurements. The pouch cells were used in their original, unmodified form without any alterations to facilitate X-ray transmission. The plexiglass holder used to apply mechanical pressure to the cell contained a 2–3 mm diameter central aperture to ensure that the holder did not scatter/obstruct the X-ray beam.*

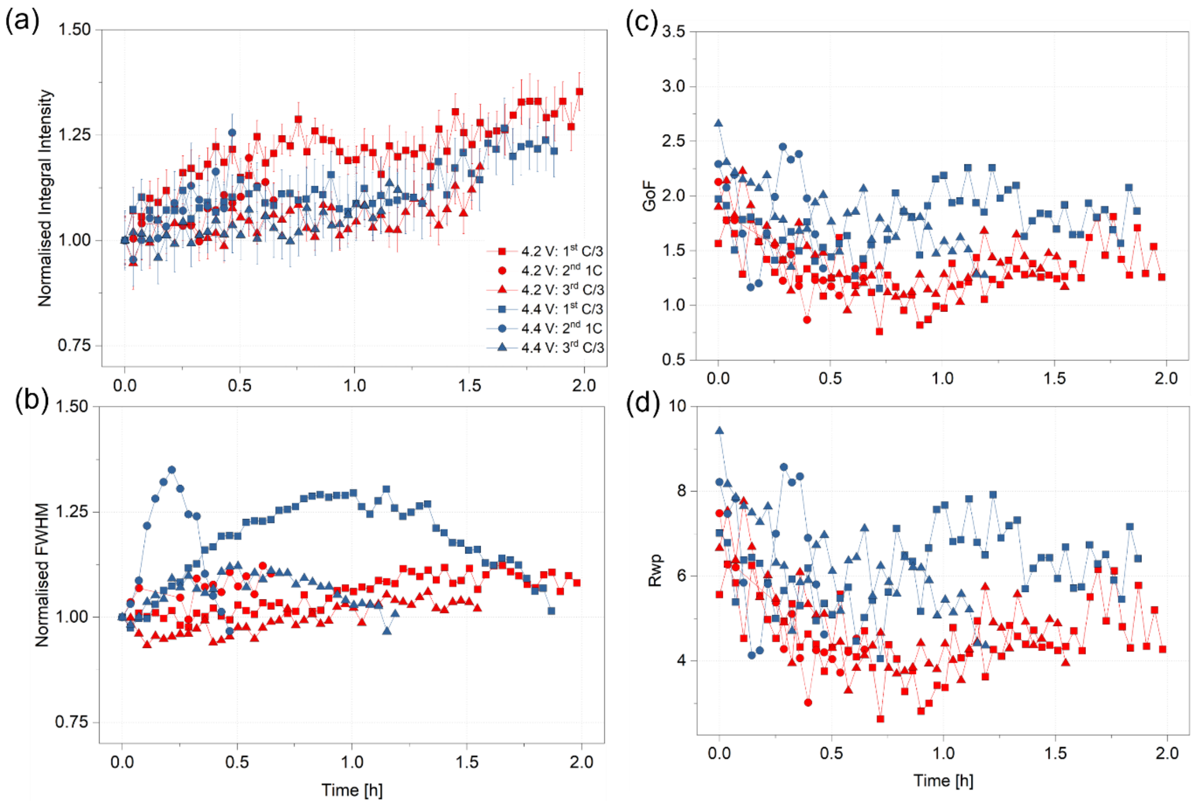

Figure S9: *Results from single-peak fitting of the (003) reflection in Regime 1 during charge for the multi-rate operando XRD data of the aged 4.2 V and 4.4 V cells, showing: (a) normalised integral intensity, (b) normalised full width at half maximum (FWHM), (c) goodness-of-fit (GoF), and (d) the weighted profile R-factor (Rwp).*

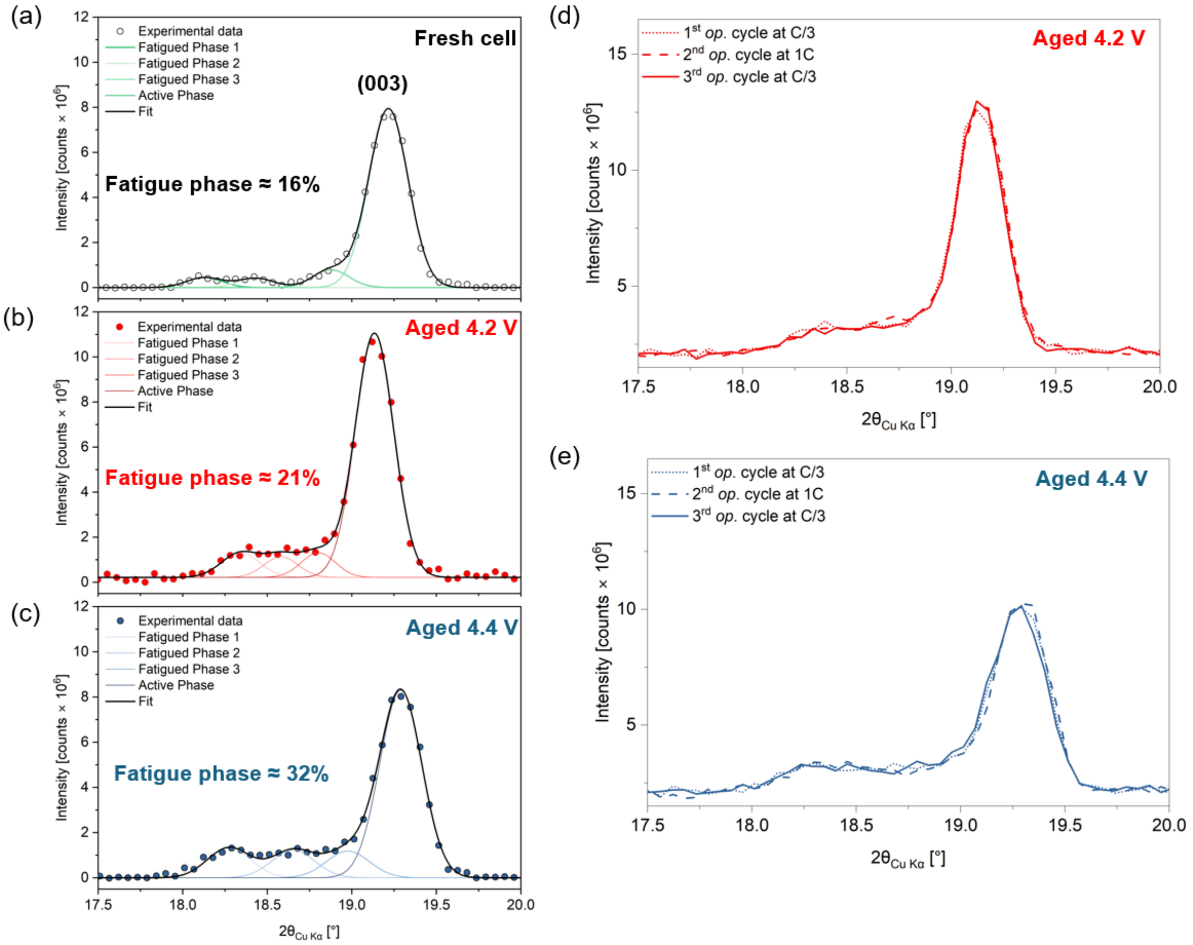

Figure S10: *Multi-peak fitting of the (003) reflection at the end of the constant-voltage (CV) step showing the contributions from the electrochemically active and fatigued phases for (a) the fresh cell, (b) the aged 4.2 V cell, and (c) the aged 4.4 V cell. The extracted fatigue phase fractions are indicated in each panel, revealing a progressive increase from the fresh cell (~16%) to the aged 4.2 V cell (~21%) and reaching a maximum for the aged 4.4 V cell (~32%). (d) Overlap of the (003) reflections recorded at the end of the CV step for different operando cycles at C/3–1C–C/3 for the aged 4.2 V cell, demonstrating that an identical state of delithiation may be attained, irrespective of C-rate, with a CV step. (e) A similar overlap is observed for the aged 4.4 V cell, confirming a consistent structural state across different cycling rates at the end of CV step.*

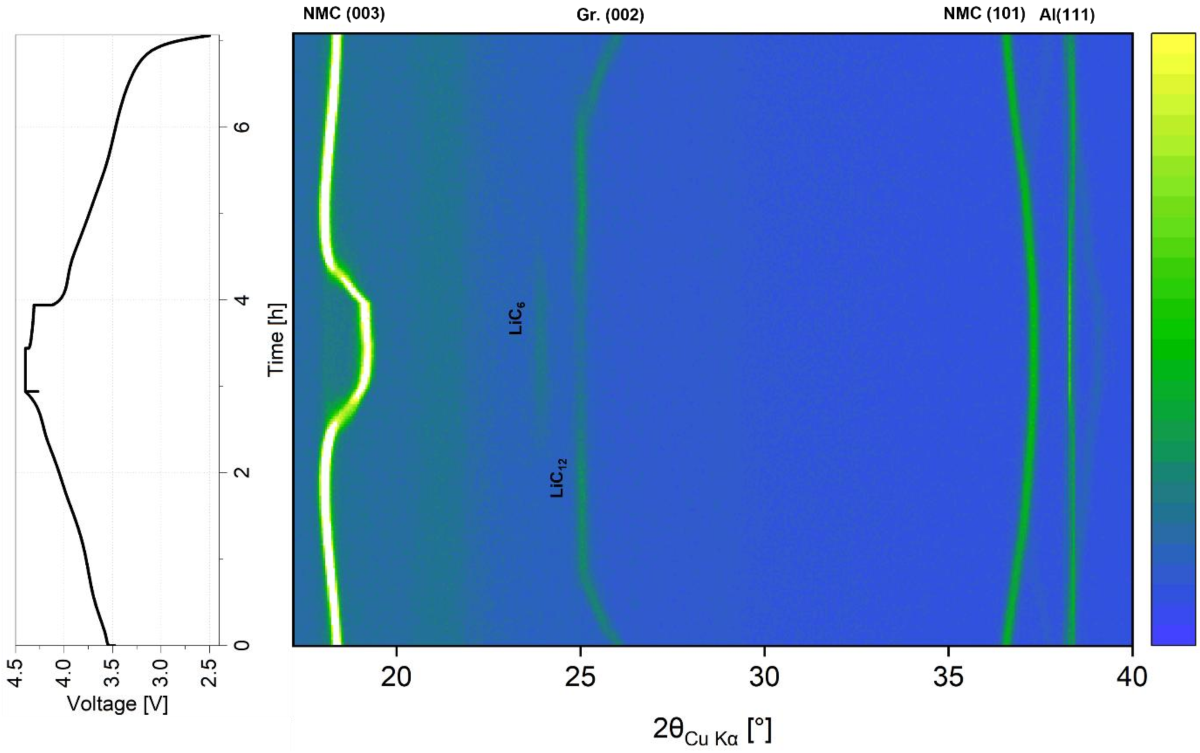

Figure S11: *Heatmap of operando XRD data collected from a fresh NMC811–graphite pouch cell after formation cycling. The corresponding voltage–time profile is shown alongside the heatmap. The operando measurement was performed at C/3 using a CC–CV charging protocol. This data is from our previous work, intended for comparative purposes.*[2]

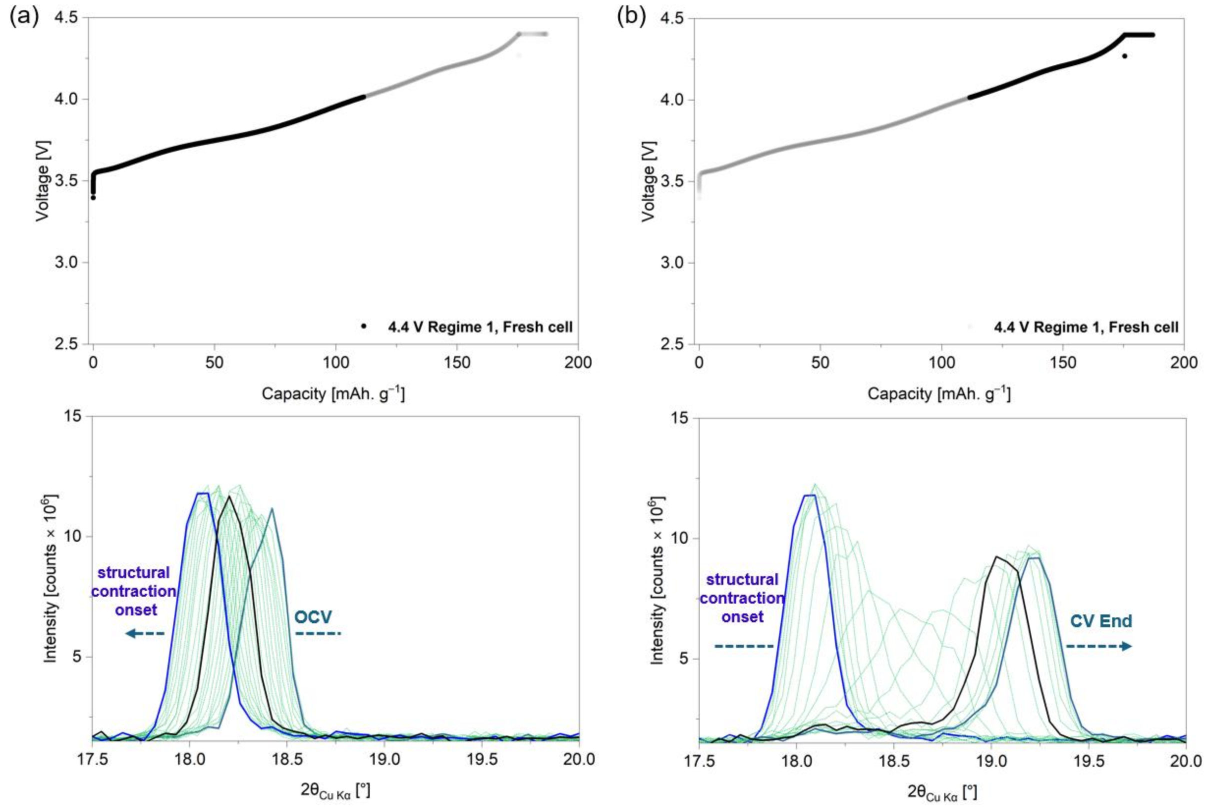

Figure S12*: Evolution of the (003) peak of fresh cell (a) from the start of charge to the onset of structural contraction (Regime 1) and (b) from the onset of structural contraction to end of the CV step (Regime 2) during the operando cycle at C/3 rate in the 2.5–4.4 V window. The corresponding voltage vs. capacity profiles are shown at the top, where the portions of charge corresponding to Regime 1 are highlighted in a darker shade of black, and the portions corresponding to Regime 2 are highlighted in a lighter shade.*

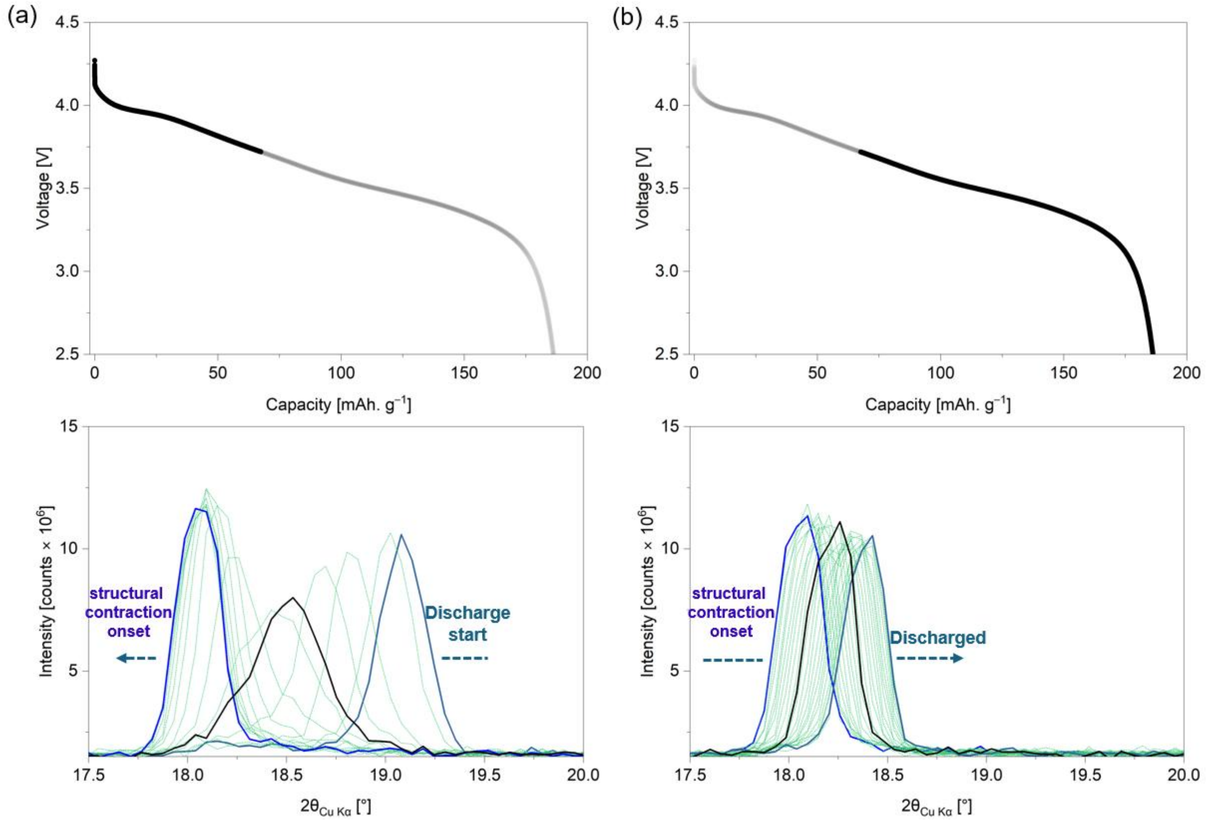

Figure S13: *Evolution of the (003) peak of fresh cell (a) from the start of discharge to the onset of structural contraction (Regime 1) and (b) from the onset of structural contraction to the discharged state (Regime 2) during the operando cycle at C/3 rate in the 2.5–4.4 V window. The corresponding voltage vs. capacity profiles are shown at the top, where the portions of discharge corresponding to Regime 1 are highlighted in a darker shade of black, and the portions corresponding to Regime 2 are highlighted in a lighter shade.*

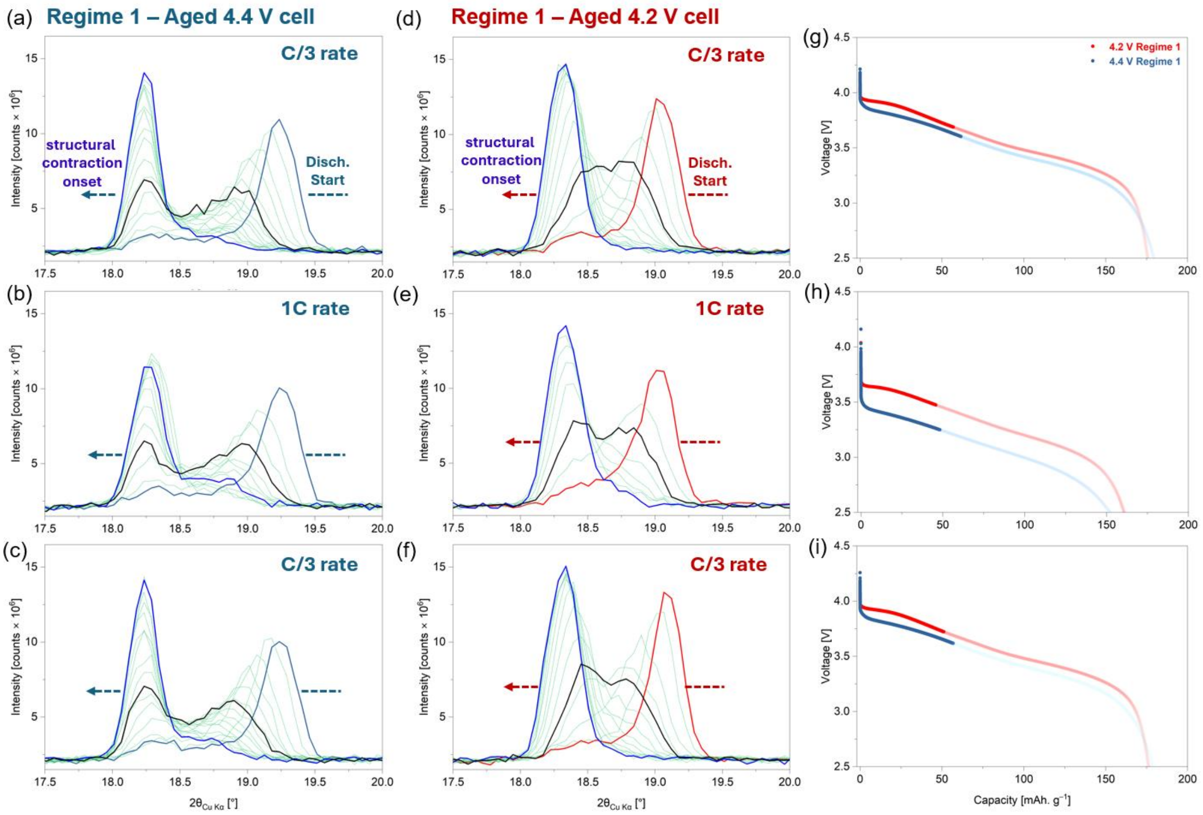

Figure S14: *Evolution of the (003) reflection from the start of discharge to the onset of structural contraction (Regime 1) for (a–c) the aged 4.4 V cell and (d–f) the aged 4.2 V cell during three consecutive operando cycles at C/3, 1C, and C/3 rates. The intermediate XRD scan showing pronounced phase separation, evidenced by clear splitting of the (003) peak, is highlighted with a solid black line. (g–i) Corresponding voltage vs capacity profiles, where the portions of discharge associated with Regime 1 are highlighted in darker shades of blue and red, and those associated with Regime 2 are shown in lighter shades of the same colours.*

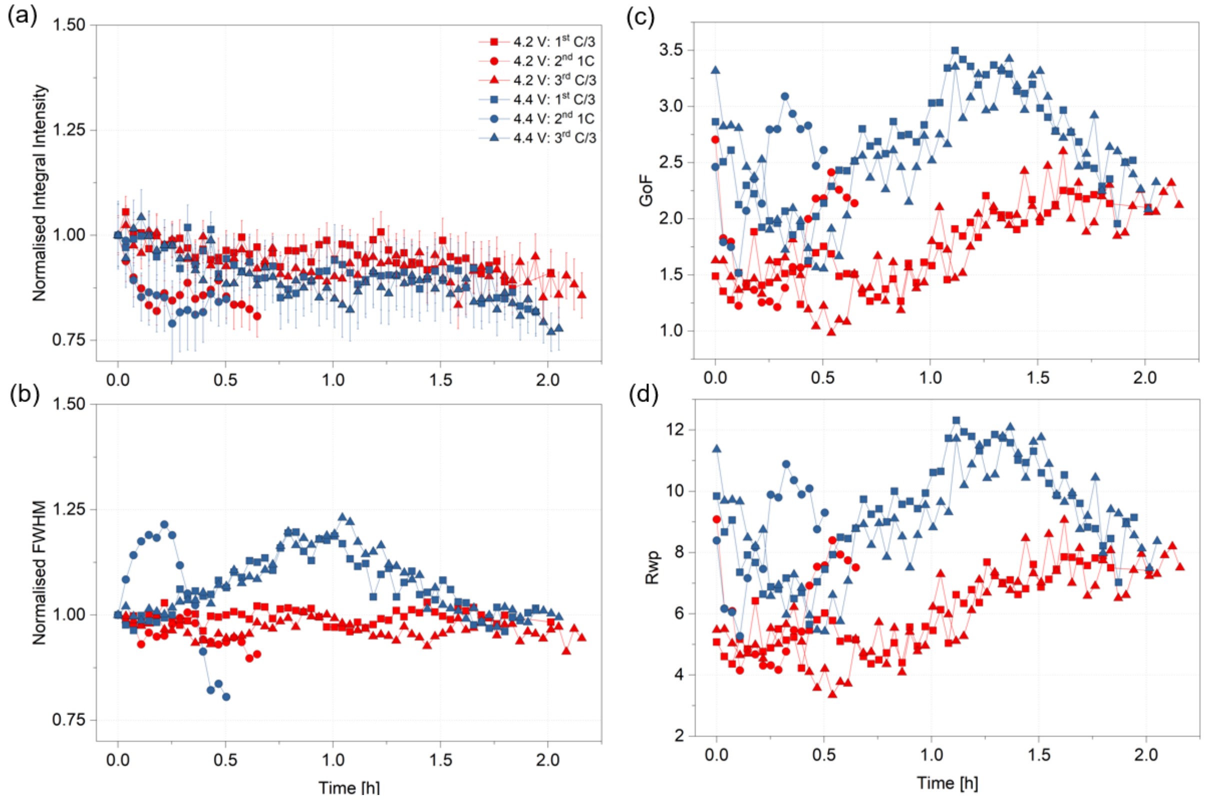

***Figure S15**: Results from single-peak fitting of the (003) reflection in Regime 2 during discharge for the multi-rate operando XRD data of the aged 4.2 V and 4.4 V cells, showing: (a) normalised integral intensity, (b) normalised full width at half maximum (FWHM), (c) goodness-of-fit (GoF), and (d) the weighted profile R-factor (Rwp).*

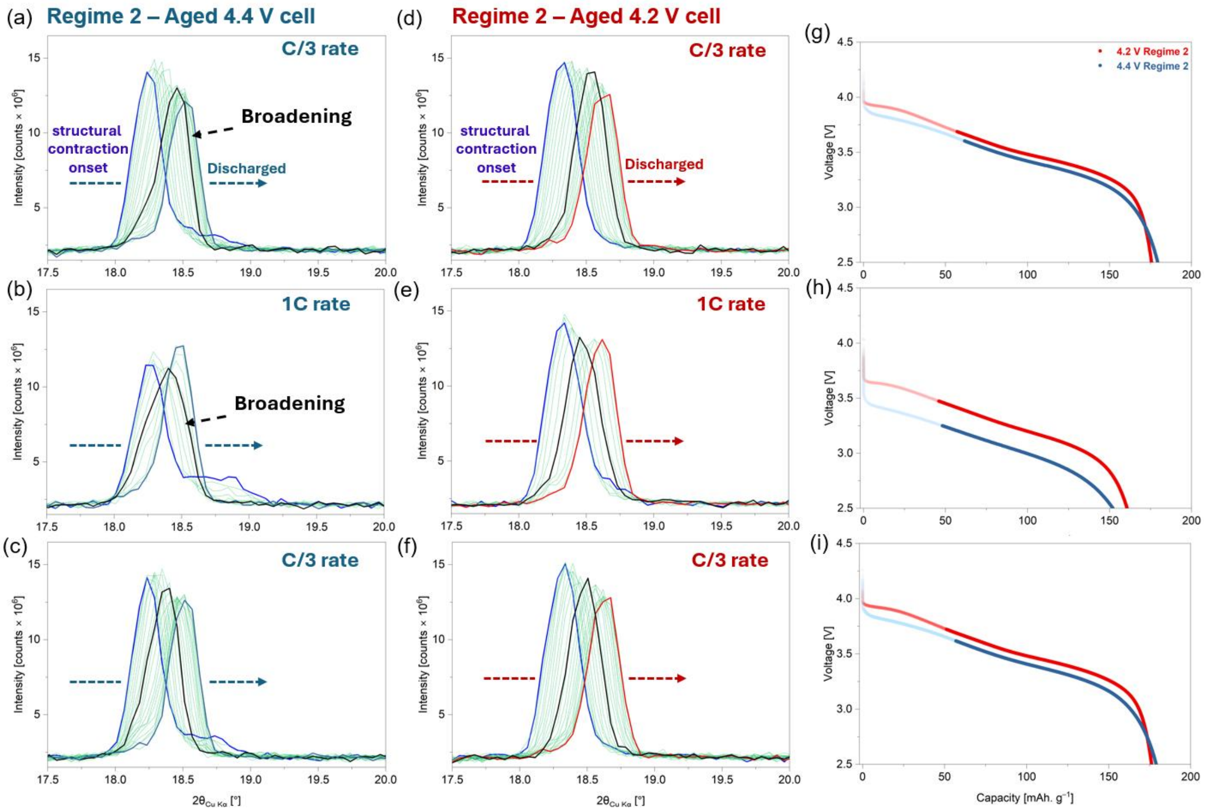

Figure S16: *Evolution of the (003) reflection from the onset of structural contraction to the fully discharged state (Regime 2) for (a–c) the aged 4.4 V cell and (d–f) the aged 4.2 V cell during three consecutive operando cycles at C/3, 1C, and C/3 rates. The intermediate XRD scan, shown as a solid black line, highlights pronounced peak broadening in the aged 4.4 V cell, whereas the aged 4.2 V cell shows minimal change in peak width. (g–i) Corresponding voltage vs capacity profiles, where portions of discharge associated with Regime 1 are highlighted in darker shades of blue and red, and those associated with Regime 2 are shown in lighter shades of the same colours.*

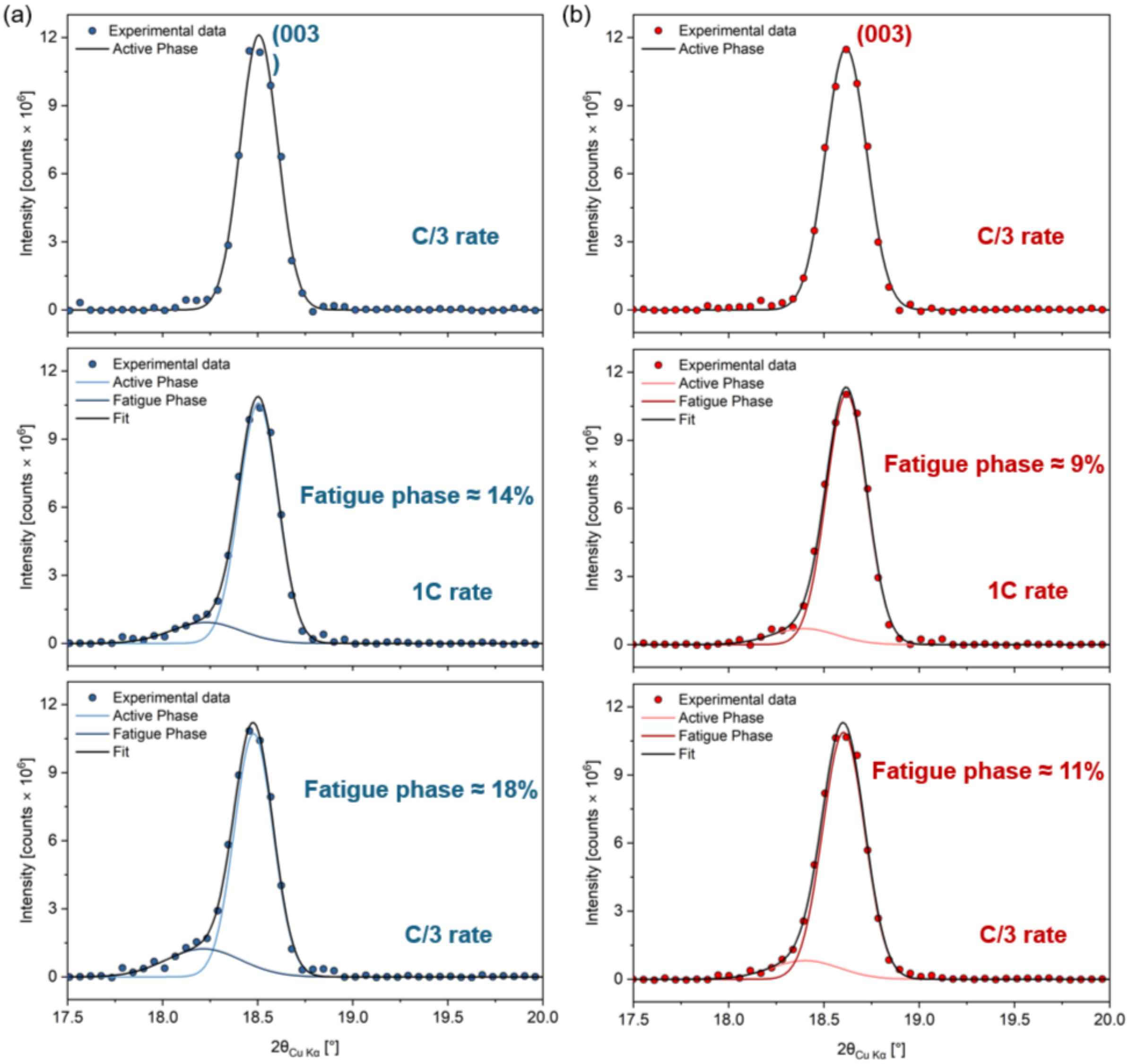

Figure S1*7*: *Peak fitting of the (003) reflection recorded at the open-circuit voltage (OCV) following successive operando cycles at C/3, 1C, and C/3 rates for (a) the aged 4.4 V cell and (b) the aged 4.2 V cell. The extracted fatigue phase fractions are indicated in the corresponding panels and reveal a progressive increase with successive cycling.The extent of fatigue phase formation is consistently higher in the aged 4.4 V cell than in the aged 4.2 V cell.*

Table S1(a): *Charge and discharge capacities and the corresponding Coulombic efficiencies obtained during the first and second formation cycles, as well as the diagnostic cycle conducted after aging on the NMC811–graphite pouch cell in 2.5-4.4 V window.*

**Cycles (2.5–4.2 V)**	**Charge capacity (mAh g^{-1})**	**Discharge Capacity (mAh g^{-1})**	**Coulombic Efficiency (%)**
1st Formation Cyc.	236.7	202.7	85.6
2nd Formation Cyc.	212.6	211.5	99.4
Last Diagnostic Cyc.	177	192.9	108

Table S1(b): *Charge and discharge capacities and the corresponding Coulombic efficiencies obtained during the first and second formation cycles, as well as the diagnostic cycle conducted after aging on NMC811–graphite pouch cell in 2.5-4.2 V window.*

**Cycles (2.5–4.4 V)**	**Charge capacity (mAh g^{-1})**	**Discharge Capacity (mAh g^{-1})**	**Coulombic Efficiency (%)**
1st Formation Cyc.	240.2	225.2	93.7
2nd Formation Cyc.	224.5	223	99.3
Last Diagnostic Cyc.	179.1	198	110

Table S2: *Impedance values obtained from the fitting of the Nyquist plots for the NMC811–graphite pouch cells cycled within 2.5-4.2 V and 2.5-4.4 V after formation and cycle life test.*

Cells	R_1	R_2	R_3	R_0
AF, 2.5–4.2 V	1.35 (17)	0.903 (236)	0.733 (74)	0.144
AF, 2.5–4.4 V	1.73 (21)	1.23 (28)	0.696 (69)	0.146
Aged, 2.5– 4.2 V	2.33 (40)	2.27 (52)	1.29 (13)	0.154
Aged, 2.5– 4.4 V	4.55 (68)	4.00 (81)	1.70 (14)	0.156

References

(1) Ogley, M. J. W.; Menon, A. S.; Pandey, G. C.; Páez Fajardo, G. J.; Johnston, B. J.; McClelland, I.; Majherova, V.; Huband, S.; Tripathy, D.; Temprano, I.; et al. Metal-ligand redox in layered oxide cathodes for Li-ion batteries. *Joule* **2025**, *9* (1). DOI: 10.1016/j.joule.2024.10.007
(2) Shah, N. A.; Páez Fajardo, G. J.; Banerjee, H.; Pandey, G. C.; Menon, A. S.; Ans, M.; Majherova, V.; Bree, G.; Bolloju, S.; Grinter, D. C.; et al. Nature of the Oxygen-Loss-Induced Rocksalt Layer and Its Impact on Capacity Fade in Ni-Rich Layered Oxide Cathodes. *ACS Energy Letters* **2025**, *10* (3), 1313-1320. DOI: 10.1021/acsenergylett.5c00324.
